# De novo basecalling of RNA modifications at single molecule and nucleotide resolution

**DOI:** 10.1186/s13059-025-03498-6

**Published:** 2025-02-25

**Authors:** Sonia Cruciani, Anna Delgado-Tejedor, Leszek P. Pryszcz, Rebeca Medina, Laia Llovera, Eva Maria Novoa

**Affiliations:** 1 Centre for Genomic Regulation (CRG), The Barcelona Institute of Science and Technology, Dr. Aiguader 88, Barcelona, 08003 Spain; 2 Universitat Pompeu Fabra (UPF), Barcelona, Spain; 3 ICREA, Pg. Lluís Companys 23, Barcelona, Spain

**Keywords:** Nanopore sequencing, Native RNA, RNA modifications, Machine learning, N6-methyladenosine, Basecalling, Training data, Single molecule resolution

## Abstract

RNA modifications influence RNA function and fate, but detecting them in individual molecules remains challenging for most modifications. Here we present a novel methodology to generate training sets and build modification-aware basecalling models. Using this approach, we develop the *m*^*6*^*ABasecaller*, a basecalling model that predicts m^6^A modifications from raw nanopore signals. We validate its accuracy in vitro and in vivo, revealing stable m^6^A modification stoichiometry across isoforms, m^6^A co-occurrence within RNA molecules, and m^6^A-dependent effects on poly(A) tails. Finally, we demonstrate that our method generalizes to other RNA and DNA modifications, paving the path towards future efforts detecting other modifications.

**Supplementary Information:**

The online version contains supplementary material available at 10.1186/s13059-025-03498-6.

## Background

RNA modifications, also referred to as epitranscriptomic modifications, are chemical alterations that occur on RNA molecules, influencing their fate and function. These modifications play critical roles in diverse biological processes, including cellular differentiation [1–3], immune responses [4, 5], cancer progression [6, 7] and sex determination [8, 9]. At the molecular level, RNA modifications can regulate gene expression [10], modulate protein translation [11], impact RNA stability [12] and affect RNA-protein interactions [13].

To date, more than 170 different RNA modifications have been described [14]. These modifications can target all four RNA bases as well as the sugar moiety, and are found in all known RNA species, including ribosomal RNAs (rRNAs), messenger RNAs (mRNAs), transfer RNAs (tRNAs) and small non-coding RNAs (snRNAs). Among these, N6-methyladenosine (m^6^A), has garnered the most attention as the most abundant internal modification in eukaryotic mRNA [15, 16], as well as due to its dynamic nature across conditions and environmental stimuli [17]. The deposition of m^6^A modifications is mediated by “writers”, which catalyse the addition of the methyl group [18, 19]. These modifications can be removed by “erasers” [20, 21], and recognized by “readers”, which selectively bind to the modified RNA [22–25]. The study of m^6^A dynamics and function has been significantly advanced by next-generation sequencing (NGS) technologies, which have facilitated transcriptome-wide mapping of m^6^A sites across different species, cell types and environmental conditions [26–32].

Nanopore direct RNA sequencing (DRS) has recently emerged as a promising alternative to NGS-based methods to comprehensively investigate the epitranscriptome [33–36]. This technology relies on measuring fluctuations in current intensity as the RNA or DNA molecules pass through the nanopores. Unlike NGS approaches, nanopore DRS enables the generation of transcriptome-wide maps of RNA modifications at the isoform level [37], facilitates the simultaneous detection of multiple RNA modification types [38, 39] and provides quantitative estimates of RNA modification levels at individual sites [40, 41]. As a result, this technology allows for the creation of transcriptome-wide epitranscriptomic maps with an unprecedented level of resolution, surpassing the capability of short-read sequencing data.

**Fig. 1:**
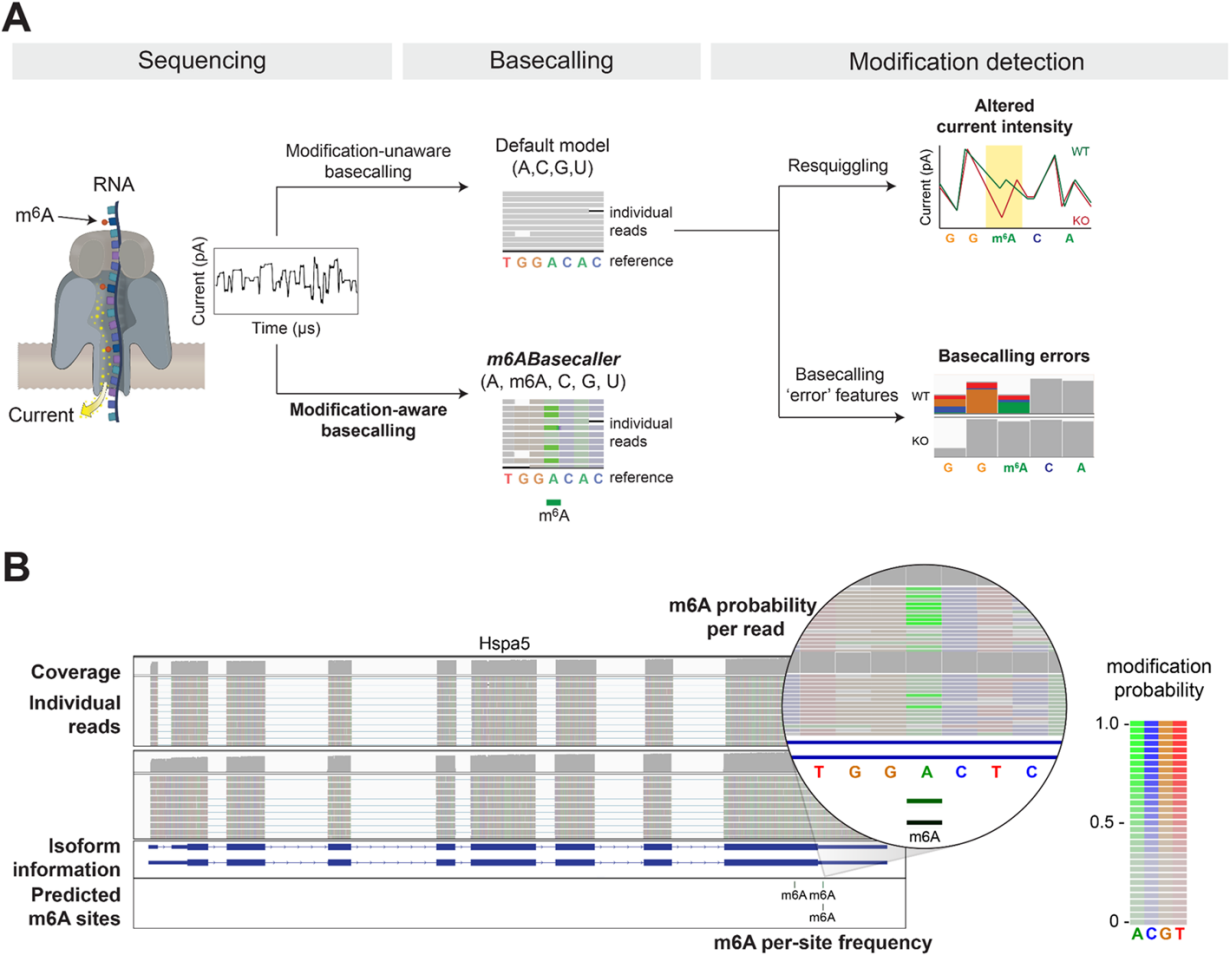
Schematic overview of the approaches that can be used to identify RNA modifications from direct RNA sequencing (DRS) data. **A** Overview of the methods used to detect modified sites from DRS data. Commonly used softwares to detect RNA modifications rely on either: i) basecalling errors that are present in a wild type (WT) but not a knockout (KO)/control condition, or ii) altered current intensities when comparing WT and KO/control conditions. All these methods use default (modification-unaware) RNA basecalling models and require extensive post-processing after basecalling steps –mapping, resquiggling, feature extraction and statistical testing– to identify modified sites. The alternative option is to use a modification-aware RNA basecalling model that predicts modifications during the basecalling step, which provides m^6^A modification predictions with single nucleotide and single molecule resolution. **B** IGV visualisation of a BAM file where reads have been basecalled using the *m*^*6*^*ABasecaller*, allowing per-read analysis of m^6^A modifications in full-length reads. BAM files have m^6^A information encoded at per-read and per-nucleotide level in the form of modification probabilities. Colouring nucleotides based on their modification probability allows simple visualisation of m^6^A-modified sites (bright green) in a transcriptome-wide fashion. A ‘predicted m^6^A site’ is defined as a position that has at least 25 reads coverage and ≥ 5% modification stoichiometry (i.e., a minimum of 2 modified reads supporting that site). A nucleotide in a read is defined as ‘modified’ if the modification probability is equal or greater than 0.5 (shown as ‘predicted m^6^A sites’) at the bottom of the IGV snapshot

In recent years, several studies have demonstrated the ability of nanopore DRS to detect a wide range of RNA modification types [42, 43], including m^6^A [44–51], pseudouridine [38, 52, 53] and inosine [54]. Two primary strategies have been mainly employed to identify RNA modifications in nanopore DRS data: (i) analysing current intensity and/or dwell time fluctuations [38, 49, 51]; and (ii) detecting modifications through the analysis of basecalling ‘errors’ [44, 55–58] (Fig. 1A). While both approaches have proven effective in detecting RNA modifications, they generally rely on comparisons with reference samples, such as those derived from knockout or knockdown conditions targeting the RNA modification ‘writer’ enzyme of interest. This dependency of such ‘paired’ conditions limits the applicability of nanopore DRS to a relatively small range of biological contexts. To address this limitation, recent methods have been developed that circumvent the requirement for ‘paired’ conditions [49]. However, these methods typically provide their final predictions at per-site level.

An alternative strategy for transcriptome-wide RNA modification detection involves de novo basecalling of modifications directly from the raw nanopore signals. In this approach, the default RNA basecalling model is replaced with a modification-aware model (illustrated in Fig. 1A). For example, the m^6^A-aware basecalling model would predict five distinct bases –A, C, G, U and m^6^A– based on the current intensity information. In contrast, the default RNA basecalling model limits its predictions to the canonical four bases – A, C, G, U.

Despite its potential, the development of modification-aware basecalling models has been hindered by significant challenges, primarily due to the scarcity of sufficient and high-quality ‘training data’ –datasets with high-confidence modification status labels. To develop a ‘successful’ basecalling model, it is essential for the model to distinguish between the modification(s) of interest and unmodified canonical bases. This requires training datasets in which the presence or absence of the RNA modification(s) at specific positions is known with certainty (i.e., the “ground truth”). However, knowing the ‘site’ alone is not sufficient; for effective training, it is critical to identify not only the positions of the modifications but also the specific reads in which the modification occurs. Such precise labeling at the per-read level is necessary to properly train the model to recognize RNA modifications in individual RNA molecules.

Here, we propose and validate a novel strategy for generating high-accuracy per-read predictions of modification status. Using this approach, we create ‘labelled’ datasets that can serve as ‘ground truth’ for training modification-aware models, and demonstrate that is applicable both to DNA and RNA molecules. We exemplify this approach by training the *m*^*6*^*ABasecaller*, which we demonstrate that it can predict m^6^A modifications de novo with single-read and with single-nucleotide resolution, achieving high accuracy and low false positive rates. We demonstrate that the *m*^*6*^*ABasecaller* can generate transcriptome-wide maps of m^6^A modifications across datasets from various species and sequencing devices, in real-time as the reads are being sequenced, without requiring knockout or control conditions. Furthermore, we show that *m*^*6*^*ABasecaller* enables the collection of m^6^A modification information at the isoform level, provides reproducible and accurate estimates of m^6^A modification stoichiometry, With this resolution, we can characterize the m^6^A modification co-occurrence within individual reads, and characterise the relationship between m^6^A presence and poly(A) tail lengths, among other features (Fig. 1B). Finally, we demonstrate that our approach is adaptable to other types of RNA and DNA modifications, applicable across diverse species, and compatible with updated RNA004 chemistries. Overall, this work provides a novel framework for generating high-quality labelled training datasets and for training modification-aware base-calling models for an expanded range of modifications. This advancement enables the direct and simultaneous analysis of multiple post-transcriptional regulatory layers within individual RNA molecules.

## Results

### Training an m^6^A-aware RNA basecalling model using synthetic constructs

Methods to detect RNA modifications in DRS datasets have typically relied on the identification of increased base-calling ‘errors’ and/or altered current intensities at the RNA modified sites. Whilst these approaches have proven useful to detect diverse types of RNA modifications [38, 44–54, 59, 60], they suffer from important caveats: (i) they require extensive manipulation and pre-processing of the signal data before being able to detect RNA modifications (basecalling, mapping, feature extraction, aggregation of features per-site, resquiggling and/or statistical testing) [61], ii) they suffer from stoichiometry biases (unmodified reads are preferentially resquiggled) [38]; iii) they require minimum coverage of ∼30–50 reads to detect a site as modified [59]; iv) they often require a minimum modification stoichiometry (~ 10–20%) to detect a site as modified [59], and (v) they often suffer from significant numbers of false positives [59].

To overcome these limitations, here we sought to build a modification-aware basecalling model that would predict m^6^A modifications de novo from raw FAST5 reads, with single-nucleotide and single-molecule resolution (Fig. 1A). To this end, we first employed synthetic RNA *‘curlcake’* constructs [44] to train a basecalling model, which contain RNA modifications in all possible 5-mer contexts. More specifically, we used *curlcakes* that were either 100% m^6^A-modified (all As had been replaced for m^6^A) or 0% modified as ‘training sets’. Our results showed that *curlcake*-trained m^6^A-aware basecalling model correctly reported modified A nucleotides as m^6^A, and unmodified bases as A, when tested with fully unmodified or fully modified reads (Additional File 1: Figure S1A), with very high per-read and per-site accuracy and very low false positive and false negative rates. However, when tested on partially modified reads that were not fully modified at all sites (only some As had been replaced for m^6^A, see *Methods*) we noticed that this model predicted all As in a given read ‘chunk’ as being either unmodified or modified (Additional File 1: Figure S1A). These results suggest that a basecalling model trained with fully modified (100%) and unmodified (0%) synthetic reads learns to predict whether signal ‘chunks’ (long stretches of RNA), rather than bases, originate from modified or unmodified reads. Consequently, these models cannot predict whether a single position is modified or not, which is the typical biological scenario. Moreover, we noticed that a model trained solely on synthetic reads (*curlcakes*) didn’t have the ability to basecall native reads from human, mouse or yeast (data not shown), likely due to the lack of biological sequence complexity of the *curlcake* sequences, despite covering all 5-mers [62].

In order to obtain a model that would be applicable to transcriptome-wide scenarios, we then opted to train a new model with m^6^A-modified and unmodified *curlcake* RNA molecules, but this time also adding unmodified yeast and mouse reads from respective m^6^A mutants (see Additional File 2: Table S1 for a full list of trained models used in this work). This model was able to basecall reads across the yeast transcriptome, solving the limitation of the previous model in terms of covering sufficient sequence complexity, but similarly to the previous model, it predicted all As in a given read chunk being as all modified or all unmodified (Additional File 1: Figure S1B). Thus, we concluded that synthetically-generated 100%-modified in vitro transcribed datasets were not adequate for training modification-aware basecalling models, because such models learnt to predict whether all or none bases were modified in a given ‘chunk’.

### Generation of high-confidence in vivo per-read labelled data to train RNA basecalling models

We then reasoned that a better approach to train a modification-aware RNA basecalling model would be to use in vivo data, with and without the modification of interest. A major limitation to use in vivo data to train basecalling models, however, is the lack of per-read modification information to adequately ‘label’ the training set at per-read level as either ‘modified’ or ‘unmodified’. In other words, in in vivo datasets, we don’t know a priori which reads and positions in the wild type condition are modified and which ones are unmodified, due to the substoichiometric nature of RNA modifications in vivo (Additional File 1: Figure S2).

**Fig. 2:**
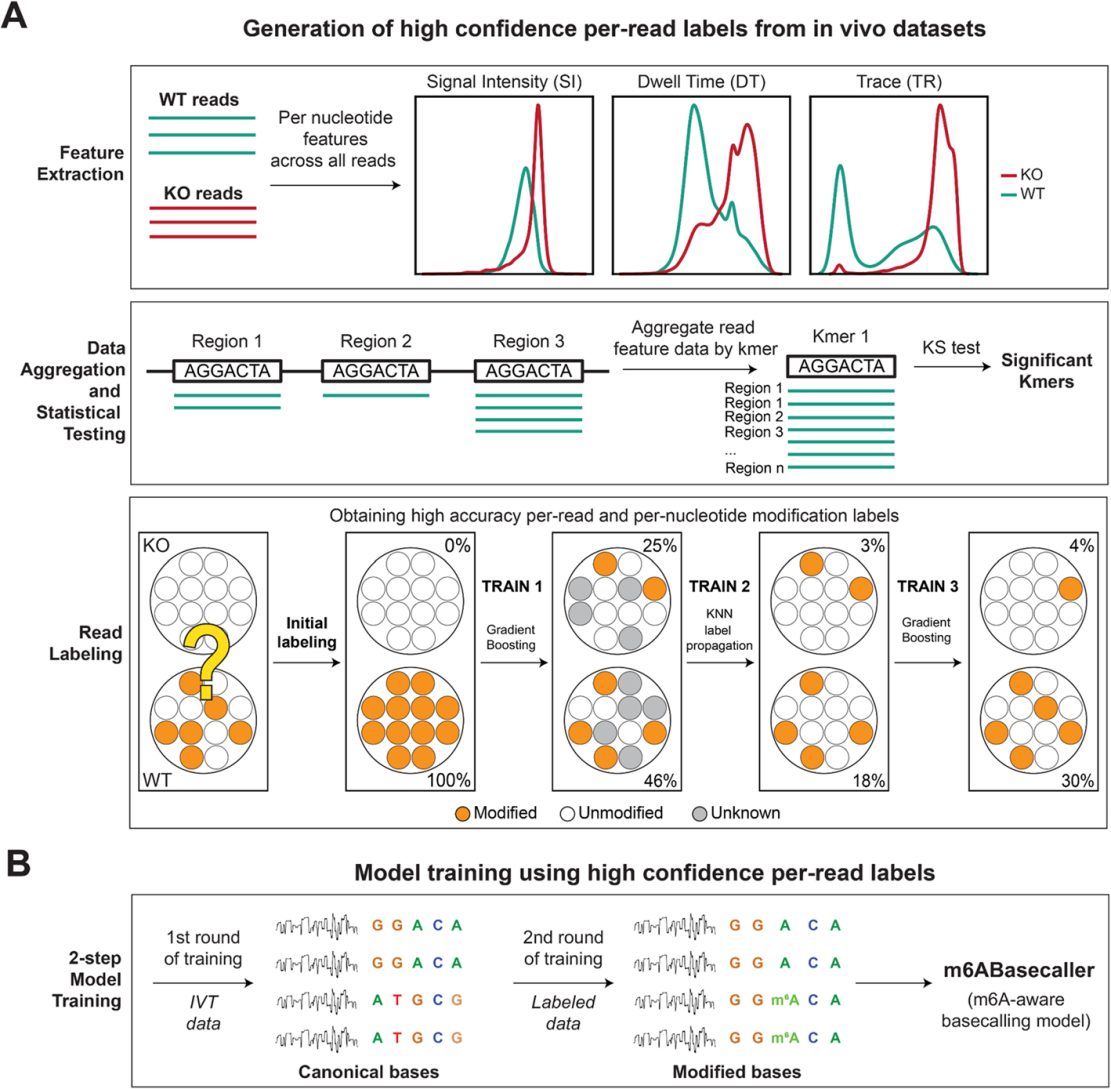
Methodology to obtain a training dataset with high-confidence RNA modification status labels, implemented in *NanoRMS2,* used to train a modification-aware basecalling model.** A** Schematic representation of steps performed to obtain high-confidence labels based on the modification status of all reads included in the training dataset. First, a set of 9 features are retrieved for every base from every read. Then, the features are aggregated for each 7-mer from the entire genome/transcriptome (balancing the number of reads between the two samples and across the reference positions). Significant 7-mers are then identified by KS-test for the two most informative features. Reads are labelled as modified or unmodified for each significant 7-mer using 50% of data (training set) in 3-step procedure as follows: i) Gradient Boosting classifier is trained assuming all KO reads as unmodified and all WT reads as modified and predicting all reads from training set either as modified or unmodified; ii) reads with low confidence prediction are marked as unknown, and label propagation (with KNN kernel) is used to label them; and iii) final (Gradient Boosting) classifier is trained with labelled training data and all reads from the test data are predicted as unmodified or modified. All these steps are performed by the *NanoRMS2* software. **B** Using the high confidence labels obtained using the procedure depicted in panel A, a modification-aware basecalling model can be trained with reads labelled as modified or unmodified, in a 2-step procedure: i) in a first step, only unmodified reads are used to train a canonical basecaller, ii) in a second step, this model is refined to call also modified bases. The second training step can be restricted to specific k-mers that are reported by *NanoRMS2*

To overcome this limitation, we hypothesized that we could use bioinformatic predictions to ‘label’ the wild type reads, which could then be used to train the model. To this end, we developed *NanoRMS2*, a new software that can produce high-confidence per-read labels of modification status, to then use as training sets for a basecaller. *NanoRMS2* builds upon *NanoRMS* [38], and relies on the extraction of raw signal features (signal intensity, dwell time and trace) to capture differences between modified and unmodified bases (Fig. 2A, upper panel). Briefly, *NanoRMS2* requires two samples: (i) a sample in which the modification of interest is present, although not necessarily at high stoichiometries (e.g., a wild type sample); and ii) a sample in which the modification of interest is not found, or its levels are very low (e.g., a knockout sample, a knockdown sample or an in vitro transcribed transcriptome [63, 64]). *NanoRMS2* then extracts 9 features for every base and from every read, namely: (i) signal intensity (SI), (ii) Modification Probability (MP), (iii) Dwell-Time at the position 0 (DT), (iv) Dwell-Time 10 bases upstream (DT10), (v) Trace values for the reference nucleotide (TR) and (vi-ix) Trace values all canonical bases (TA, TC, TG and TT, for A, C, G and T/U, respectively). Then, it aggregates the features by k-mer, and assesses which k-mers are significantly different between the two given conditions (e.g. wild type and knockout/control condition) using a Kolmogorov-Smirnov (KS)-test (Fig. 2A, middle panel). For each significant k-mer, *NanoRMS2* splits all reads equally into training and testing sets, trains a classifier (Gradient Boosting) and predicts modified/unmodified reads in the training set (Training #1).

A key difference that distinguishes *NanoRMS2* from other softwares that train classifiers based on nanopore signal-related features, such as *NanoRMS* [38], is that it does not keep these predictions as ‘final’, but rather, it only retains the ‘high confidence’ predictions, treating the rest as ‘unknown’. It then performs a semi-supervised learning step (Label Propagation) to predict the labels of the ‘unknown’ reads (Training #2). Finally, it trains a second classifier (Gradient Boosting) using a re-labelled training set (Training #3), and this trained classifier is the one that will be finally used to predict modified/unmodified nucleotides in each individual read from the testing set (Fig. 2A, lower panel). *NanoRMS2* repeats this procedure for each k-mer.

### Modification-unaware basecalling models trained with synthetic reads improve feature separation between modified and unmodified reads

The choice of datasets that are used to train a base-calling model greatly affects the quality of the trained models; similarly, the features extracted from modified and unmodified datasets, also strongly affect the performance of the trained models, as not all features capture equally well modification information across k-mers. In this regard, previous works have shown that *Trace* (TR) –the modification probability emitted by the basecaller– is one of the features that best separates modified and unmodified reads, compared to other features such as *Signal Intensity* (SI) and *Dwell Time* (DT), at least in the case of pseudouridylation (Y) and 2’-O-methylation (Nm) [38]. However, whether this observation holds true in the case of m^6^A modifications is unknown.

To address this question, we examined how *Trace*, *Signal Intensity* and *Dwell Time* separated m^6^A-modified and unmodified reads. Surprisingly, *Trace* (TR) did not separate these two populations well (Additional File 1: Figure S3A). We hypothesised that this observation could be caused by the fact that m^6^A modifications were present in the native RNA molecules that were used to train the ‘default’ canonical RNA basecalling model (Fig. 1A). In this scenario, the model would have learnt to predict m^6^A modifications as unmodified As, consequently leading to poor differences in *Trace* scores between m^6^A-modified and unmodified reads. Notably, we found this to be also true in the case of 5mC and 6mA DNA modifications (Additional File 1: Figure S3B).

We then reasoned that a canonical basecalling model –which predicts unmodified bases– trained only with unmodified RNA bases should show increased *Trace* differences between A and m^6^A nucleosides. To this end, we trained a modification-unaware ‘canonical’ RNA basecalling model using in vitro transcribed RNA –which is devoid of modifications– as training data (‘IVT model’) (see Additional File 2: Table S1). Similarly, we trained a canonical DNA basecalling model using PCR-amplified DNA as training data (‘PCR model’). We found that both these models maximised the difference in *Trace* features between unmodified and modified bases, while not affecting other features such as *Signal Intensity*, for both RNA (Additional File 1: Figure S3C,D) and DNA (Additional File 1: Figure S3E,F), thus improving the binning of modified and unmodified reads, and confirming our hypothesis that the default RNA and DNA basecalling models have incorrectly ‘learnt’ to predict an m^6^A modification as and ‘A’. Therefore, our results showed that *Trace* is a strong feature separating m^6^A-modified and unmodified reads, but base-calling models trained with datasets devoid of the RNA modifications (e.g. in vitro transcriptomes) must be used to obtain *Trace* features that will strongly differentiate the two populations.

### m6A-modification-aware models can be trained using in silico-’labelled’ in vivo data

We then proceeded to train an m^6^A modification-aware basecalling model. To this end, *NanoRMS2* was used to extract features from publicly available DRS datasets (HEK293T wild type and METTL3 KO), which had been previously base-called using our *in-house* trained canonical ‘IVT-model’ (Additional File 2: Table S1) to maximise *Trace* differences between m^6^A-modified and unmodified reads (Additional File 1: Figure S3). *NanoRMS2* was then used to obtain the final set of high-confidence m^6^A-modified and unmodified reads, (Fig. 2A), which were in turn used to train an m^6^A-modification aware basecalling model, which we refer to as ‘*m*^*6*^*ABasecaller*’ (Fig. 2B, see also Additional File 2: Table S1). Thus, the *m*^*6*^*ABasecaller* is capable of base-calling 5 different bases (A, C, G, U and m^6^A) in individual reads, completely de novo, with single nucleotide resolution, without the need of paired conditions, without the need of minimum coverage per site, and without the need of minimum stoichiometry per-site (Fig. 1B), thus offering the possibility to study the function and dynamics of m^6^A modifications in individual native RNA molecules.

A key feature of the method presented here is that the model is trained with biological data, previously ‘labelled’ as “modified” or “unmodified” using in silico approaches (in this case, using labels predicted by *NanoRMS2*). This approach has several key advantages relative to training using synthetic sequences: firstly, we do not pre-bias our training to specific k-mers (e.g. DRACH)[65]; rather the sequence context is found by *NanoRMS2* and is then used to train the model. Secondly, this approach does not require the motif to be known, thus making the method applicable to virtually any DNA/RNA modification of interest –the method actually reveals the motif, even if unknown–. Thirdly, the method is not limited to RNA/DNA modifications that can be chemically synthesised, as the method is trained with naturally existing RNA/DNA modifications that are present in native molecules. On the other hand, the trained basecalling model will in turn also show some key advantages, including: i) the model will be able to accurately recognize the RNA modification of interest in the biological context of interest; ii) the modification detection occurs truly *“*de novo”, during the basecalling step (which can be performed even during sequencing, if ‘live basecalling’ option is enabled); iii) the detection of each modification is performed independently from other reads, and thus is not affected by the read coverage at that given position; iv) no post-processing is needed to identify modifications, reducing the computational burden typically associated with modification analysis (e.g., ‘feature extraction’ or ‘resquiggling’ steps).

### *m6ABasecaller* predicts m6A modifications with high accuracy and low false positive rates

We then proceeded to benchmark the *m*^*6*^*ABasecaller* with synthetic m^6^A-modified datasets, in which the ground truth is known. We should note that in our method, each base is basecalled with an associated modification probability value (modProb, see *Methods*), and a high modification probability will indicate that the base in question is modified (m^6^A-modified in the case of *m*^*6*^*ABasecaller*). To determine the optimal modification probability threshold at which a base should be considered as ‘modified’, we examined the distribution of modification probabilities in fully unmodified (0% m^6^A-modified) and fully m^6^A-modified (100%) ‘curlcake’ [44] sequenced using DRS (Additional File 1: Figure S4A). Our results showed that the median modification probabilities of unmodified (median modProb = 0.0) and m^6^A-modified (median modProb = 0.7) bases were significantly distinct, thus allowing us to accurately identify whether a nucleotide of a specific read is modified or not by using a specific modProb threshold.

We then examined whether similar results would be observed in biological contexts. To this end, we examined the distribution of modification probabilities in transcriptome-wide in vitro transcribed (IVT) samples (“in vitro transcriptomes” [64, 66]), in two independent replicates (see Additional File 2: Table S2). IVT samples (Additional File 1: Figure S4B) constitute essential controls for methods that are meant to be applied transcriptome-wide, as they will reveal whether a given method will predict high numbers of false positives in in vivo contexts [66], which is typically a major problem in the field of RNA modifications, leading to controversies on whether a given modification is present in a specific population of RNAs or not [67–71]. Using the criteria of the Youlden Index, we determined that modProb = 0.1 is the ‘optimal’ threshold that maximised True Positives (TPs) while minimising the number of False Positives (FPs) (Additional File 1: Figure S4C). We should note that this threshold can be varied depending on whether the user wants to be more or less conservative with regards to the number of False Positives. For example, higher thresholds (e.g., modProb = 0.5) can be chosen to reduce the number of false positives. Notably, using this threshold, *m*^*6*^*ABasecaller* only predicted 15 replicable m^6^A sites in “in vitro transcriptomes”, which corresponds to a False Positive Rate (FPR) of 0.0012% (Additional File 2: Table S3).

Finally, we examined how the choice of modProb threshold affected per-site m^6^A modification stoichiometry predictions. To this end, we used in silico mixtures of modified and unmodified reads to achieve different stoichiometry ranges (0%, 25%, 50%, 75% and 100%), and stoichiometry was calculated using different modProb thresholds (0.5, 0.1 and 0.01) (Additional File 1: Figure S4D). The *m*^*6*^*ABasecaller* showed very good correlation between observed and expected stoichiometries for all modProb thresholds, being 0.1 the optimal threshold in terms of balance of FP and TP, as previously noted.

### *m6ABasecaller* accurately predicts m6A modifications in individual reads both in vitro and in vivo

**Fig. 3:**
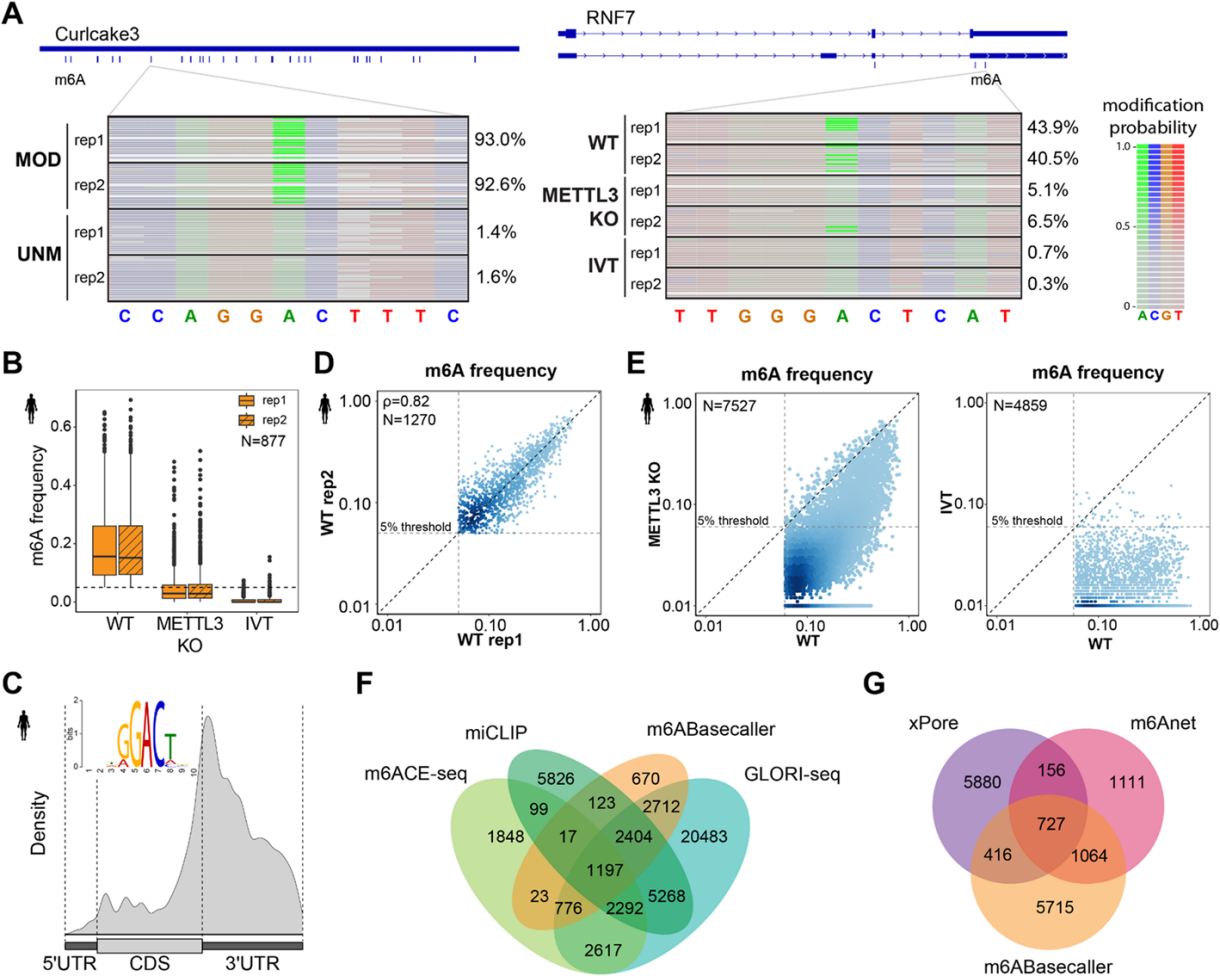
*m*^*6*^*ABasecaller* predicts m^6^A in synthetic and native RNA molecules, and shows strong overlap with predicted m^6^A sites using orthogonal methods.** A** In the left panel, IGV snapshot of individual reads base-called with *m*^*6*^*ABasecaller*. The reads are centered at a known m^6^A site, both for synthetic m^6^A-modified curlcake reads (‘MOD’, upper panel) and their unmodified counterpart (UNM, lower panel). In the right panel, reads mapping to human RNF7 gene are shown, in HEK293T WT and METTL3 KO samples, as well as for reads from in vitro transcribed (IVT) human samples. Each row represents a distinct RNA read, and each base from each read has been coloured according to its modification probability. See also Additional File 1: Figure S5 for additional IGV snapshots. **B** Boxplot of per-site m^6^A frequencies in two independent replicates of: (i) HEK293T WT, (ii) HEK293T METTL3 KO and (iii) IVT human transcriptome. Only m^6^A sites detected in WT (≥ 5% m^6^A modification frequency) and with at least 25 reads of coverage in all replicates have been included in this analysis (*N* = 877). The horizontal dashed line indicates the 5% threshold for a site to be identified as “m^6^A-modified”. **C** Metagene plot depicting the distribution of m^6^A sites detected in HEK293T WT samples (*N* = 1270). In the upper left corner, the motif obtained with MEME analysing 20 nt sequence context of all replicable sites in HEK293T WT (*N* = 1270) is shown. See also Additional File 1: Figure S6A,B for metagene plots in additional species. **D** Replicability of m^6^A modification frequency in sites with modification frequency greater or equal than 5% and minimum of 25 reads of coverage in both HEK293T WT replicates (Spearman’s *ρ* = 0.82). Dashed vertical and horizontal lines depict the 5% threshold applied to a m^6^A site to be called. Both axes are log_10_ scaled. **(E)** Scatterplot comparing per-site m^6^A frequencies in modified sites identified in HEK293T cells in WT and upon METTL3 KO (left panel)**,** and in WT compared to IVT control (right panel). Dashed vertical and horizontal lines depict the 5% threshold applied to a m^6^A site to be called. Both axes are log_10_ scaled. **F** Overlap between m^6^A sites detected by *m*^*6*^*ABasecaller* in HEK293T cells and m^6^A sites predicted using Illumina-based orthogonal methods (m6ACE-seq, miCLIP and GLORI-seq) in HEK293T cells. To provide a comparison across all methods that is independent of sequencing coverage, the set of predicted m^6^A sites by each orthogonal method was reduced to those m^6^A sites for which there was a sufficient coverage in the nanopore DRS dataset, i.e., only m^6^A sites with minimum of 25 reads coverage in the DRS dataset were included in the comparative analysis. **G** Comparison of m^6^A sites predicted by *m*.^*6*^*ABasecaller* and those predicted by other nanopore-based methods (xPore and m6Anet), ran on the same set of reads from HEK293T cells (pooled 2 replicates, see Additional File 2: Table S3)

We examined the performance of the *m*^*6*^*ABasecaller* in synthetic ‘curlcake’ constructs that were either unmodified (0% m^6^A) or fully modified (100% m^6^A) [72]. Our results showed that *m*^*6*^*ABasecaller* predicted m^6^A sites in biologically relevant DRACH sequence contexts with high accuracy and reproducibility (Fig. 3A, see also Additional File 1: Figure S5), and with very low amounts of false positives (only 0.3-0.5% were predicted as modified in the same exact sequence context and sites in the unmodified curlcake control sequences).

We then examined the performance of the *m*^*6*^*ABasecaller* in in vivo datasets. To this end, we ran the *m*^*6*^*ABasecaller* on publicly available HEK293T wild type (WT) DRS datasets, as well as on HEK293T METTL3 knockout (KO) and in IVT whole transcriptome human DRS datasets as negative controls (Fig. 3A, right panel, see also Additional File 2: Table S2 for full list of DRS datasets used in this work). The *m*^*6*^*ABasecaller* (modProb > 0.5) predicted 6,664 (rep1, 1.2 M reads) and 1,625 (rep2, 400 K reads) m^6^A-modified sites in the HEK293T WT samples, with a median per-site modification stoichiometry of 15.6% and 15.1%, respectively (Fig. 3B, see also Additional File 2: Table S4). We observed a sharp decrease in the m^6^A modification levels upon METTL3 KO on the same set of sites, which showed a median per-site modification frequency of 2.8% (rep1) and 2.9% (rep2), respectively (Fig. 3B). This decrease in stoichiometry was even more evident in IVT samples, with median modification frequency per-site of 0.0% (rep1) and 0.0% (rep2) suggesting that METTL3 KO samples are not completely devoid of m^6^A modifications in their mRNAs, in agreement with recent works [73]. The estimated median m^6^A modification frequency in vivo was not significantly affected by the modification probability threshold of choice (0.5 or 0.1), neither in WT HEK293T cells nor METTL3 KO cells (Additional File 2: Table S3, see also Additional File 2: Table S6 for predicted per-site m^6^A modification stoichiometries).

### *m6ABasecaller* predicts m6A sites and their stoichiometry levels in diverse species with high replicability and low false positive rates

We then assessed the performance of the algorithm on different species, including human (HEK293T cells), mouse (mES cells) and zebrafish (4 h-post-fertilization embryos). The *m*^*6*^*ABasecaller* predicted 7,922, 8,511 and 8,711 m^6^A-modified sites in human (see Additional File 2: Table S5), mouse (listed in Additional File 2: Table S7) and zebrafish (listed in Additional File 2: Table S8) datasets, respectively (see also Additional File 2: Table S3). The predicted m^6^A sites were largely located around the stop codon and 3’UTR region, and corresponded to DRACH motifs, in all 3 species examined (Fig. 3C, see also Additional File 1: Figure S6A,B), in agreement with previous works using orthogonal methodologies to map m^6^A modifications [26, 27, 74]. Of note, we observed that the metagene distribution of m^6^A sites did not present a 5’UTR peak that is sometimes observed when using orthogonal antibody-based methods, which has been reported to be caused by cross-reactivity of the anti-m^6^A antibodies with m^6^Am modifications [75]. In this regard, the *m*^*6*^*ABasecaller* is trained to recognise bases that change upon METTL3 KO, and consequently, it is not susceptible to confounding m^6^A with m^6^Am.

We then examined whether the m^6^A sites predicted by the *m*^*6*^*ABasecaller* were replicable in independent biological replicates that were independently sequenced, in both mouse and human samples, both qualitatively and quantitatively. Our analyses showed that the overlap of m^6^A-modified sites predicted by the *m*^*6*^*ABasecaller* across biological replicates was very high (83–89% overlap, see Additional File 1: Figure S6C,D). Similarly, the per-site predicted modification stoichiometry was highly replicable across biological replicates (Spearman’s *ρ* = 0.82–0.87, see Fig. 3D and Additional File 1: S5E). We should note that the correlation of m^6^A modification frequencies across biological replicates improved with increased sequencing coverage, as well as when only m^6^A sites with higher read coverage were included in the analysis (Additional File 1: Figure S6F).

To further validate the *m*^*6*^*ABasecaller*, we examined whether knockout (KO) of METTL3 or METTL14 led to a loss of m^6^A sites identified in the WT samples. Comparative analysis of WT and METTL3 KO samples revealed that 7,369 (98%) and 4,114 (89%) m^6^A sites showed decreased m^6^A frequencies upon METTL3 KO in HEK293T and mES cells, respectively (Fig. 3E, see also Additional File 1: Figure S7A,B). Moreover, the median per-site m^6^A frequency decreased from 15.1–15.6% to 2.8–2.9% upon METTL3 KO in HEK293T cells (Additional File 1: Figure S7C, see also Additional File 2: Table S4) and from 16.4–18.8% to 4.7–7.8% upon METTL3 and METTL14 KO, respectively, in mES cells (Additional File 1: Figure S7D, see also Additional File 2: Table S4).

### m^6^A sites predicted by *m*^*6*^*ABasecaller* are largely supported by orthogonal methods

We then assessed whether the m^6^A sites predicted by *m*^*6*^*ABasecaller* were also identified using orthogonal methods such as GLORI-seq [29], miCLIP [30] and m6ACE-seq [45]. To this end, we examined the overlap between m^6^A sites predicted by* m*^*6*^*ABasecaller* in HEK293T cells and those predicted by other orthogonal techniques in the same cell line. Only m^6^A sites for which we had sufficient coverage (> 25 reads coverage) in the HEK293T DRS dataset were kept for downstream comparisons: 37,749 sites for GLORI-seq, 17,226 sites for miCLIP, and 8,869 sites for m6ACE-seq (Additional File 2: Table S9, see also *Methods*). These sites were then compared to the 7,922 sites detected by *m*^*6*^*ABasecaller*. Our analyses revealed that 91% of m^6^A sites predicted by the *m*^*6*^*ABasecaller* were also predicted by one or more orthogonal methods (Fig. 3F). More specifically, 89% of the sites identified by *m*^*6*^*ABasecaller* were also identified by GLORI-seq (Additional File 1: Figure S8A). We then examined the correlation between the stoichiometries predicted by the two methods (GLORI-Score vs predicted stoichiometry by *m*^*6*^*ABasecaller*) in replicable sites (*N* = 2023), finding a strong correlation (Spearman’s *ρ* = 0.73, *p* < 2.2 e^−16^) (Additional File 1: Figure S8B). A more modest overlap was observed with miCLIP (47%) and m6ACE-seq (25%), respectively (Additional File 1: Figure S8C,D). We should note, however, that the overlap of sites between Illumina methods themselves was also low, with only 7.6% of predicted m^6^A sites detected by all 3 Illumina-based methods (Additional File 1: Figure S8E, see also Additional File 2: Table S10).

Lastly, we examined the overlap between m^6^A sites predicted by *m*^*6*^*ABasecaller* and those predicted by other Nanopore tools, namely xPore [45] and m6Anet [49], on the same publicly available HEK293T DRS datasets [45]. Our results showed that only 14% and 22% of the sites detected by *m*^*6*^*ABasecaller* were also identified using xPore and m6Anet, respectively (Fig. 3G). However, m6Anet predicted fewer predicted m^6^A sites (3,058) compared to *m*^*6*^*ABasecaller* (7,922), possibly due to a minimum modification stoichiometry that is required by this method to identify a site as ‘modified’ (see next section). Notably, 58% of the sites predicted by m6Anet were also predicted by *m*^*6*^*ABasecaller*.

### *m6ABasecaller* shows accurate stoichiometry prediction even at low stoichiometries

We proceeded to evaluate the performance of *m*^*6*^*ABasecaller* at predicting m^6^A modifications at low modification frequencies (i.e., lower than 20%). Indeed, our work suggests that the median m^6^A modification frequency of human samples is ~ 15% (Fig. 3B, see also Additional File 2: Table S3), which is lower than any of the initial synthetic mixes at varying stoichiometries tested (Additional File 1: Figure S4D, minimum tested was 25%). Of note, several methods for detecting RNA modifications in DRS datasets are known to under-perform at stoichiometries below 20% [42, 44, 51, 56, 76], which paradoxically, seems to be the scenario of in vivo m^6^A-modified sites in human and mouse mRNAs, at least for the cell lines and samples examined in this work.

To this end, we generated new synthetic mixes with known m^6^A modification stoichiometries by subsampling different proportions of reads from the 100% and 0% m^6^A-modified ‘curlcakes’, generating datasets that contained 0%, 6.25%, 12.5%, 25%, 50% and 100% m^6^A-modification stoichiometries (datasets available for reproducibility purposes, see *Methods*). We then analyzed the synthetic mixtures using both *m*^*6*^*ABasecaller* and m6Anet, and compared the accuracy and performance of the two methods at GGACU motifs (*N* = 14), DRACH motifs (*N* = 194) and KGACY motifs (DRACH motifs without any other A in the 5-mer except for the central one, *N* = 44), using the 0% m^6^A-modified dataset as a means to determine the number of false positives. Firstly, we examined the ability of each method to identify sites as ‘modified’, even at low stoichiometries. Our results revealed that, while both softwares showed low numbers of false positives (0 in the case of *m*^*6*^*ABasecaller* and 1 in the case of m6Anet, see Additional File 2: Table S11), *m*^*6*^*ABasecaller* outperformed m6Anet in terms of sensitivity (Additional File 1: Figure S9A, see also Additional File 2: Table S11) for all stoichiometries and sequence contexts tested, except for one (100% m^6^A-modified, KGACY context, see Additional File 2: Table S11). Overall, our results show that from a per-site perspective, *m*^*6*^*ABasecaller* was more specific, sensitive and accurate than m6Anet for almost all stoichiometries and sequence contexts analysed (Additional File 2: Table S11).

We then examined the accuracy of stoichiometry prediction for *m*^*6*^*ABasecaller* and m6Anet using the same synthetic mixes. In the case of *m*^*6*^*ABasecaller*, modification stoichiometry was defined as the number of reads that showed modProb > 0.1, whereas in the case of m6Anet, modification stoichiometry was defined as the mod_ratio that is reported per-site by the algorithm. We should note that m6Anet did not report any sites at stoichiometries lower than 50% (Additional File 1: Figure S9A,B). Thus, to make the results comparable, we chose to include the mod_ratio for all sites, for each modification stoichiometry. We observed that m6Anet overestimated the modification frequency for 6.25%, 12.5% and 25% stoichiometry datasets, reaching a better accuracy at higher stoichiometries (Additional File 1: Figure S9C,D). We reasoned that this might explain the stringent threshold that m6Anet uses to define a site as ‘modified’, which appears to come at the expense of not reporting low-stoichiometry sites, which are often the biological scenario, and also would explain the ~ twofold decrease in the number of reported m^6^A sites, compared to *m*^*6*^*ABasecaller*, when analyzing the same DRS dataset (Fig. 3G). On the contrary, *m*^*6*^*ABasecaller* did not show any false positives nor false negatives, but showed slight underestimation of modification stoichiometry at higher stoichiometries (Additional File 1: Figure S9C,D). Overall, our results showed that *m*^*6*^*ABasecaller* is both more accurate and sensitive at calling m^6^A sites and at predicting frequency at lower stoichiometries, which are more similar to those that are also found in biological samples.

Finally, to further validate our predicted global modification stoichiometries, we examined if the overall predicted m^6^A abundances predicted by the *m*^*6*^*ABasecaller* would be consistent with the previously reported values, obtained with orthogonal techniques [77]. To this end, we calculated the %m^6^A/A in both human (HEK293T) and mouse (mESC) samples, finding that *m*^*6*^*ABasecaller* predicted an m^6^A/A values of ~ 0.2% (Additional File 2: Table S12). Notably, these values are in a similar range to those previously reported m^6^A/A ratios estimated using LC–MS/MS (0.15–0.6%, according to different studies, and reviewed in [77].

### *m6ABasecaller* recapitulates modification stoichiometry variations upon STM2457 treatment

**Fig. 4:**
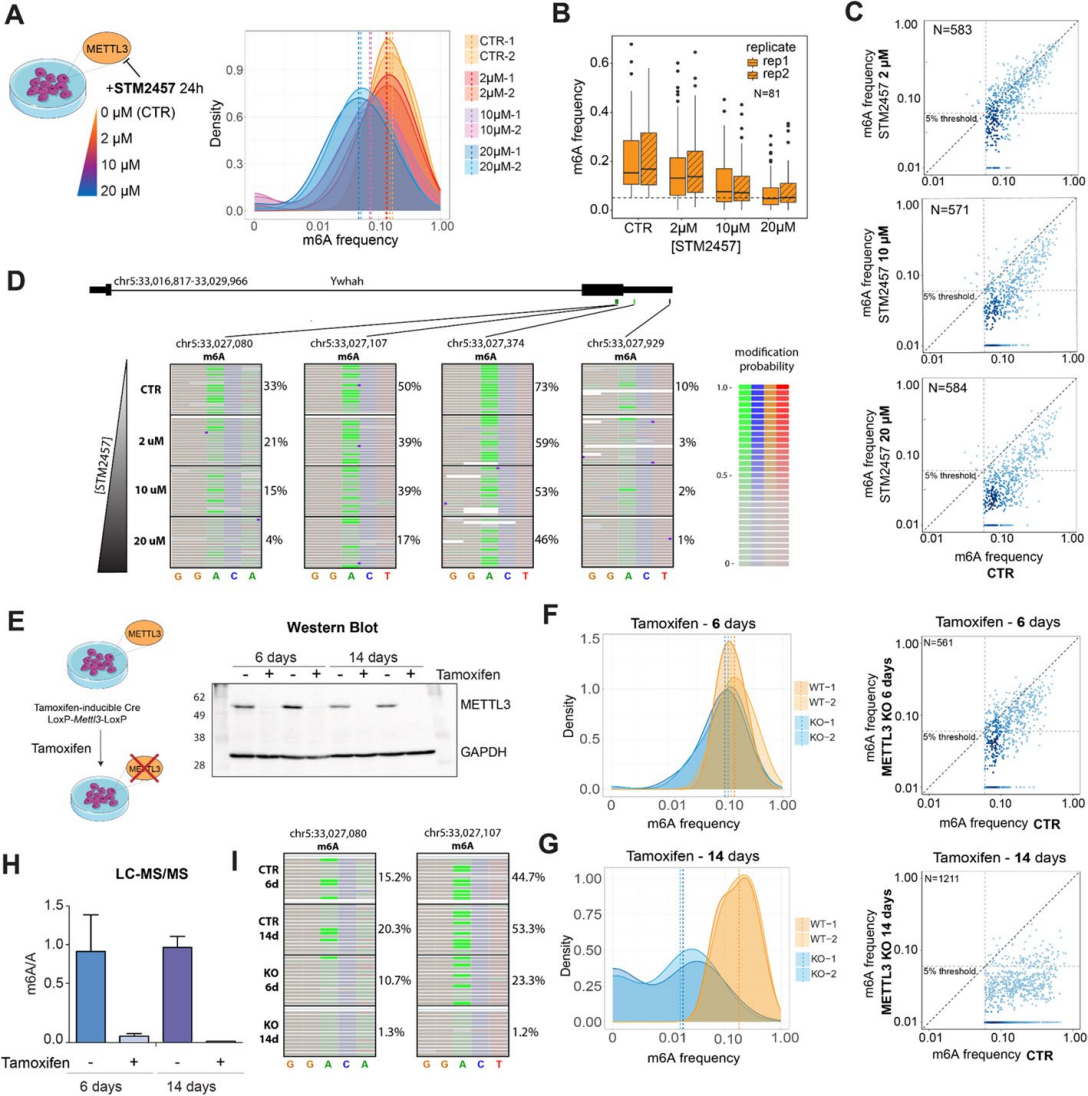
*m*^*6*^*ABasecaller* accurately predicts m^6^A modification frequencies transcriptome-wide. **A** Density plots of m^6^A modification frequencies in mESC samples treated with different concentrations of STM2457 inhibitor (0, 2, 10 and 20uM). Results are shown for two independent biological replicates. Dashed vertical lines indicate the median m^6^A frequency of each sample. **B** Boxplot of the Distribution of the m^6^A frequency at different concentrations of STM2457 in sites with more than 25 reads of coverage in all replicates of all conditions (*N* = 81). **C** Scatterplots depicting the per-site m^6^A modification frequency in untreated samples (CTR) relative to STM2457-treated samples (2 µM, upper panel; 10 µM, middle panel; 20 µM upper panel). A gradient from light to dark blue shows the increase in density of data points in the plot. Dashed diagonal black line indicates the x = y line in frequencies. Grey vertical and horizontal dashed lines indicate the 5% m6A frequency threshold used to identify a site as ‘m^6^A-modified’. Axes are log-scaled. **D** IGV snapshot depicting the decrease of m^6^A modified reads with increasing concentration of STM2457. The number of reads containing bright green (high probability of m^6^A) diminishes with the increase of STM2457 dosage. The purple dots represent base insertions. **E** On the left side, a scheme depicting the generation of tamoxifen-inducible METTL3 KO mESC cell lines is shown. On the right, a Western Blot image depicting the loss of METTL3 upon tamoxifen treatment for 6 days (2 replicates) and 14 days (2 replicates), compared to MetOH-treated cells (CTR) for 6 and 14 days, respectively. GAPDH was used as loading control. **F**,**G** In the left panels, density plot distribution of the m^6^A frequency in the pooled replicates of mES cells treated with tamoxifen (KO) for 6 days (F, *N* = 57 sites) or 14 days (G, *N* = 213 sites), compared to those treated with MetOH (CTR). In the right panels, scatterplots depicting the modification frequency at m^6^A sites detected in the pooled control samples (CTR) compared to the corresponding frequency in pooled samples treated with tamoxifen for 6 days (**F**) or for 14 days (**G**). A gradient from light to dark blue shows the increase in density of data points in the plot. Dashed diagonal black line indicates the x = y line in frequencies. Grey vertical and horizontal dashed lines indicate the 5% m6A frequency threshold used to identify a site as ‘m^6^A-modified’. Axes are log-scaled. For F and G, a pseudocount of 0.001 was added to all values to allow logarithmic scaling of the values. **H** Quantification of m^6^A levels in polyA + RNA from mESC cells treated with tamoxifen for 6 days or 14 days. m^6^A/A is computed as the ratio of m^6^A area vs A area in LC–MS/MS results. 6-day and 14-day tamoxifen treatment led to a ~ 15X and 90X reduction in m^6^A levels compared to untreated control samples, respectively (**I**) IGV snapshot depicting the decrease of m^6^A modified reads with increasing duration of tamoxifen treatment. The number of reads containing bright green (high probability of m^6^A) diminishes with the METTL3 inhibition and longer tamoxifen exposure

We then assessed the ability of the *m*^*6*^*ABasecaller* to capture variations in m^6^A modification frequencies in biological samples in a quantitative manner. To this end, we sequenced polyA-selected RNA from mESC treated with increasing concentrations of STM2457 (0, 2, 10 and 20 µM), a well-characterised inhibitor of METTL3 [78]. Our analysis revealed that the m^**6**^A modification frequency observed transcriptome-wide progressively decreased with increasing concentrations of the inhibitor (Fig. 4A). Indeed, untreated samples showed 15.2–16.7% median m^6^A modification frequency, and decreased to 13.0–13.6%, 7.5–7.1% and 4.7–5.1% upon 2, 10 and 20 µM of STM2457 treatment, respectively (Fig. 4B-D, see also Additional File 2: Table S4). Notably, the per-site m^6^A modification stoichiometry values were highly replicable across biological replicates (Additional File 1: Figure S10A,B), with predicted m^6^A sites matching the DRACH motif (Additional File 1: Figure S10C), mostly located in the stop codon and 3’UTR regions of coding transcripts (Additional File 1: Figure S10D), in agreement with previous reports [26, 27, 74]. Overall, our results show that the *m*^*6*^*ABasecaller* can robustly identify quantitative changes in m^6^A modification stoichiometry in a transcriptome-wide fashion and in a replicable manner, in addition to qualitative changes transcriptome-wide.

### *m6ABasecaller* reveals incomplete loss of m6A in inducible METTL3 KO systems

To further validate the *m*^*6*^*ABasecaller*, we generated an inducible METTL3 KO cell line by introducing 2 LoxP sequences upstream of the METTL3 exon 2 and downstream of the METTL3 exon 4 –as described in [79]– into a mER-Cre-mER mESC line, which constitutively expresses Cre recombinase fused to a mER domain, inducing its translocation to the nucleus upon tamoxifen treatment, thus making the knockout system tamoxifen-inducible (Fig. 4E, see also *Methods*). To determine the duration of tamoxifen treatment required to observe a complete loss of METTL3 protein, mESC were treated with tamoxifen for 1, 3, 6 and 14 days, and METTL3 protein levels were quantified using Western Blotting, showing that 6 and 14 days –but not 1 or 3 days– of tamoxifen treatment led to a complete loss of METTL3 protein in all examined clones (Fig. 4E). Thus, all subsequent analyses were performed using 6-day tamoxifen-treated (which we refer to as ‘METTL3 KO’) or vehicle-treated (‘CTR’) mES cells.

We then sequenced METTL3 KO and CTR mESC cells in biological duplicates using nanopore DRS (Additional File 1: Figure S11A), and predicted m^6^A-modified sites in individual reads using *m*^*6*^*ABasecaller*. To our surprise, we only observed a modest reduction in the median m^6^A modification frequencies when comparing CTR (~ 13% median m^6^A frequency) and METTL3 KO samples (~ 10.5% median m^6^A frequency) (Fig. 4F, see also Additional File 2: Table S3), Moreover, only 41% of m^6^A sites identified in CTR samples (*n* = 231) disappeared upon tamoxifen treatment –i.e., fell below the 5% modification frequency threshold– despite these conditions leading to a complete loss of METTL3 protein (Fig. 4E).

Puzzled by these results, we hypothesised that the loss of METTL3 might not lead to an immediate loss of m^6^A modifications in mRNA molecules, and that our observations could be explained by the presence of m^6^A modifications that were previously deposited in mRNAs that have not yet been degraded. We reasoned that if this were the case, the 14-day tamoxifen-treated samples should show significantly less m^6^A than the 6-day tamoxifen-treated cells. To test this, we sequenced polyA-selected RNA from 14-day tamoxifen-treated and matched CTR mESC cells in biological duplicates (Additional File 1: Figure S11B) and analysed the DRS data using *m*^*6*^*ABasecaller*, using same settings and parameters than previously employed for 6-day treated/untreated samples. Notably, this time we observed a drastic reduction in the median m^6^A modification frequencies when comparing untreated (~ 18% median m^6^A frequency) and 14-day tamoxifen-treated samples (~ 1.7% median m^6^A frequency) (Fig. 4G, see also Additional File 2: Table S4). Indeed, 91% of m^6^A sites identified in CTR samples (*n* = 1,106) falling below the 5% modification frequency detection threshold upon tamoxifen treatment. Thus, our results support the hypothesis that there is a delay between the loss of METTL3 protein and the loss of m^6^A in mRNAs.

To further validate the results obtained using *m*^*6*^*ABasecaller*, we examined the m^6^A modification levels in polyA-selected RNAs from 6- and 14-days tamoxifen-treated samples using Liquid Chromatography coupled to Mass Spectrometry (LC–MS/MS) [80]. This analysis revealed a 14-fold decrease in m^6^A levels in 6-day tamoxifen-treated samples, compared to CTR untreated samples (Fig. 4H). This difference was further increased upon extending the tamoxifen treatment; 14-day tamoxifen-treated showed a 90-fold decrease in m^6^A levels, compared to 14-day CTR untreated samples. We should note that the fold-change observed in LC–MS/MS was higher than in nanopore DRS, possibly due to different biases in the two technologies, such as the number of rounds of polyA selection and/or purity of the polyA selected material (data not shown). Despite the differences in absolute m^6^A levels measured by the two platforms, the relative m^6^A differences measured by LC–MS/MS overall support our model, further confirming that inducible METTL3 knockout systems require very long treatments to fully deplete the system from m^6^A modifications, and are in agreement with the results observed by *m*^*6*^*ABasecaller*, demonstrating that while m^6^A levels globally decrease upon induced METTL3 protein loss, absence of METTL3 protein does not guarantee absence of m^6^A in mRNAs, as some m^6^A-modified mRNAs seem to require additional time to disappear (Fig. 4I).

### Per-read analysis reveals direct correlation between m^6^A, polyA tailing and splicing

**Fig. 5:**
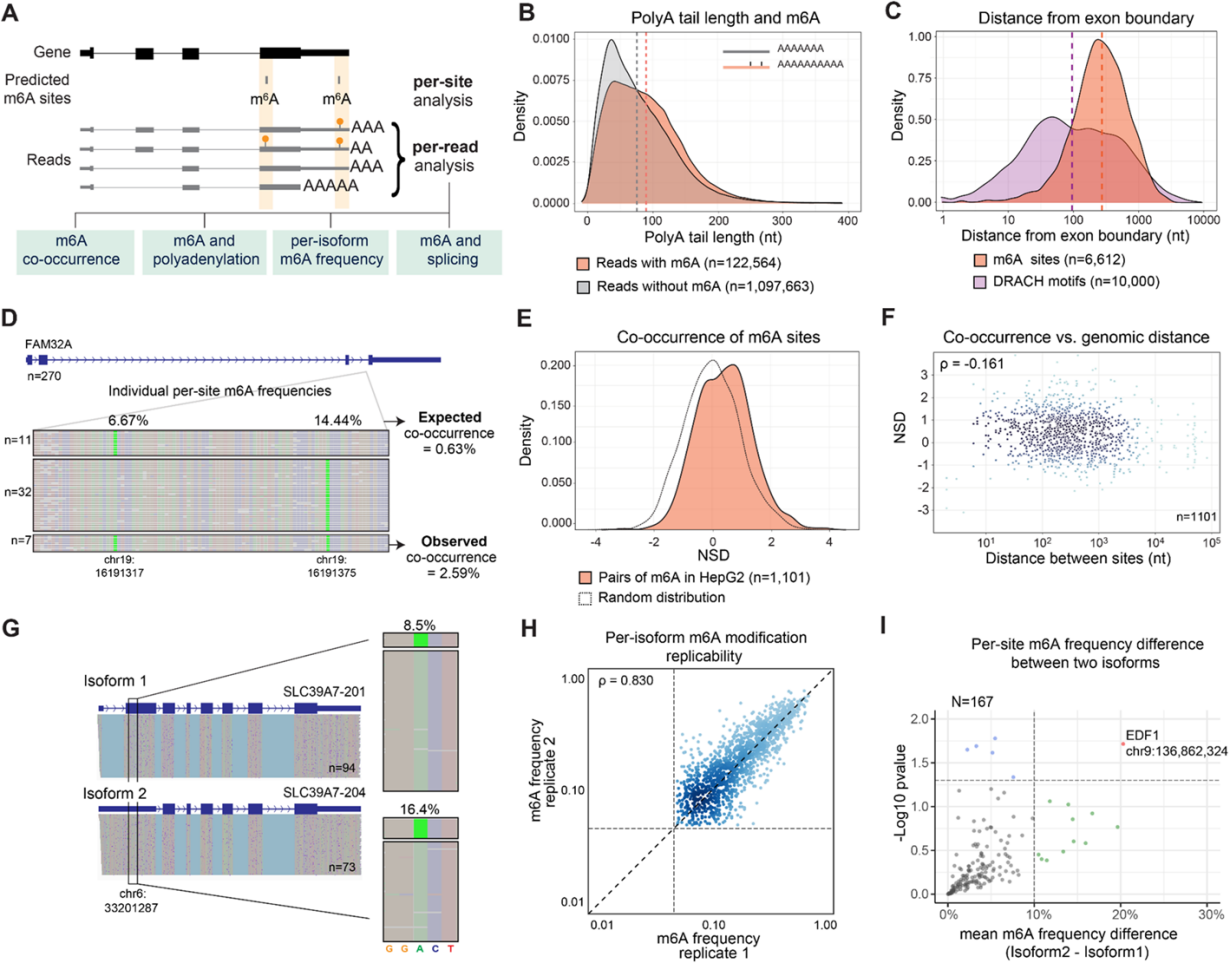
Analysis of m^6^A modifications at per-read level. **A** Schematic overview of the distinct layers of information that can be studied with per-read resolution.** B** Distribution of polyA tail lengths in reads containing at least 1 m^6^A site (orange) and reads without m^6^A (grey). Dashed line indicates the median polyA tail length of each group. All reads for which tailfindr gave a prediction of tail length > = 10 nt were included in the analysis (*N* = 1.224.173 reads; median polyA tail length: 75nt for no_m6A; 90nt for m6A-containing reads). See also Additional File 1: Figure S11 for density plots using subsets of reads for each bin. **C** Distribution of the distance between each m^6^A modification and the closest boundary of an exon (orange), compared to the distribution of the same distance calculated for a random subset of DRACH motifs in the same genes (purple). The x axis is log_10_-scaled. **D** IGV snapshot of reads mapping to FAM32A gene. Bases have been coloured according to modification probability, showing that this gene contains two m^6^A modifications at positions chr19:16,191,317 and chr19:16,191,375, depicted with bright green colour. Reads have been binned depending on whether they contain one m^6^A modified site (chr19:16,191,317), the other m^6^A modified site (chr19:16,191,375), or both sites modified. The observed and expected co-occurrence values, given the individual per-site m6A modification frequencies, are also shown. Reads that had both positions unmodified are not shown. **E** Distribution of number of standard deviations (NSD) values, which quantifies the co-occurrence of pairs of m^6^A sites (*N* = 1,101) in HepG2 cells is shown in red. As a control, a random distribution generated with the same amount of data points and the same standard deviation, centered in 0, is also shown. **F** For each pair of m^6^A sites (*N* = 1,101) analysed, the number of standard deviations (NSD) is plotted against the genomic distance (log_10_-scaled) between the two sites. Each dot represents a pair of m^6^A sites. Spearman correlation is shown. **G** IGV snapshot depicting the modification frequency at position chr6:33,201,287 in both isoforms (SLC39A7-201 and SLC39A7-204) from gene SLC39A7. Modification frequency at per-isoform level is shown. **H** Scatterplot depicting the correlation of m6A frequencies at per-isoform level. Grey vertical and horizontal dashed lines indicate the 5% m^6^A frequency threshold used to identify a site as ‘m^6^A-modified’. Axes are log-scaled. **I** One-sided volcano plot depicting isoform-specific m^6^A modification patterns. In the y axis, the mean absolute difference in ‘m^6^A modification frequencies across 2 isoforms (*N* = 167 comparisons) in two replicates is shown. To increase the statistical power of the analysis as well as the number genes for which isoform-specific m^6^A analysis was possible, all HepG2 MinION reads were pooled as one replicate (*N* = 4,741,372 reads, see Additional File 2: Table S2). HepG2 reads from a PromethION run were used as a second replicate (*N* = 6,200,572). Only reads unambiguously assigned to a given isoform were kept for the analysis

A major strength of the *m*^*6*^*ABasecaller*, compared to Illumina-based methods and other nanopore-based methods that do not have per-read resolution, is that in addition to providing m^6^A modification information at single nucleotide and single molecule resolution, it allows direct correlation between the presence of m^6^A and other post-transcriptional features that can be identified and/or measured in the same RNA molecules, such as polyadenylation or splicing. Indeed, *m*^*6*^*ABasecaller* allows investigating questions such as: i) whether m^6^A modifications co-occur simultaneously in the same read or are mutually exclusive (‘m^6^A co-occurrence’); ii) whether the presence of m^6^A affects polyA tail lengths; and iii) whether m^6^A modifications are (un)equally deposited across isoforms of a same gene (Fig. 5A).

To address these questions, we processed publicly available DRS runs from HepG2 polyA-selected RNA, generated by the Singapore consortium [81]. These runs included more than 13 million reads (Additional File 2: Table S2), thus making it possible to perform per-isoform m^6^A analyses on this dataset. The *m*^*6*^*ABasecaller* identified a total of 28,846 m^6^A sites (listed in Additional File 2: Table S13) in the pooled set of 13 million HepG2 reads. Mapped reads were then filtered to retain only full-length reads that were uniquely assigned to one isoform, which were subsequently used for per-isoform m^6^A analyses detailed below.

We then examined whether the presence of m^6^A in mRNAs globally affected polyA tail lengths. To this end, RNA molecules were binned into two groups: (i) those with one or more m^6^A modifications, and (ii) those without m^6^A modifications. We compared the polyA tail length distributions of the two groups, finding that m^6^A-containing mRNA molecules showed significantly longer polyA tail lengths (median pA tail length = 90nt) than their unmodified mRNA counterparts (median polyA tail length: 75nt) (*n* = 1.224.173 reads, Mann-Whitney-Wilcoxon test *p* < 2.2e^−16^, Fig. 5B). Notably, this difference was significant also when limiting the analysis to genes that contained m6A sites (*N* = 442,126 reads, median polyA tail length: 84nt for no_m6A, 90nt for m6A, Mann–Whitney-Wilcoxon test *p* < 2.2e^−16^, Additional File 1: Figure S12A). To ensure that this analysis was not biased by reads from a few highly expressed genes, we also examined the per-gene median polyA tail length of m^6^A-modified reads and of unmodified reads, such that each gene contributes equally, finding that the differences between the two populations were more modest, but still significant (*n* = 1507 genes, median polyA tail length: 89nt for no_m6A, 92nt for m6A, Mann-Whitney-Wilcoxon test *p* < 0.05, Additional File 1: Figure S12B).

Recent works have provided evidence for m^6^A being excluded from exon junctions [82–85]. Thus, we wondered whether the same trend would be observed in DRS datasets analysed with *m*^*6*^*ABasecaller*, as they provide both isoform and m^6^A information for each RNA molecule sequenced. To this end, we computed the distance between each m^6^A site (*n* = 6,612 sites, those that could be unequivocally assigned to a single transcript), to the closest exon start or end. As a control, the distance between randomly-selected DRACH motifs and the closest exon start or end was also computed (see *Methods*). Our analysis revealed that the distance between m^6^A sites and the closest exon boundary was significantly higher (median = 274 nt) than the one expected by chance in randomly chosen DRACH motifs (median = 95 nt) (*p*-value = 1.97e^−12^, see also Fig. 5C), in agreement with previous observations [82–84] using orthogonal methods.

### m^6^A modifications are preferentially deposited on the same mRNA molecule

mRNA molecules are typically substochiometrically modified, implying that not all molecules mapping to a given m^6^A site are m^6^A-modified. Whether m^6^A modifications are preferentially deposited in a subset of mRNA molecules (‘hyper-modified RNAs’) or uniformly placed across molecules is largely unknown, mainly due to our inability to map m^6^A modifications in individual RNA molecules. Here we exploited the single molecule resolution feature of the *m*^*6*^*ABasecaller* to address this question. To this end, we first extracted m^6^A information from full-length RNA reads that could be unambiguously assigned to a given mRNA isoform (see *Methods*). For each pair of m^6^A-modified sites present in a given isoform, we computed the ‘expected co-occurrence’ by multiplying the m^6^A modification frequencies of each of the two m^6^A-modified sites (*n* = 1,101 pairs of m^6^A sites in the HepG2 dataset). Then, we compared this value to the ‘observed co-occurrence’ (Fig. 5D, see also Additional File 1: Figure S13A), which we defined as the proportion of molecules that have both m^6^A sites modified. Finally, for each pair of m^6^A sites, we calculated the number of standard deviations (NSD) that the ‘observed co-occurrence’ deviated from the expected co-occurrence (see *Methods*). Therefore, pairs of sites with NSD > 0 co-occur more frequently than expected, whereas m^6^A pairs with NSD < 0 are preferentially deposited in distinct RNA molecules.

We examined the distribution of NSD values for all pairs of m^6^A sites transcriptome-wide, finding that the distribution of NSD values was slightly shifted towards positive values (Fig. 5E). To assess whether this shift was significant, we compared this distribution to a random distribution with same sample size and variance, but mean value of 0, finding that the two distributions were significantly different (Mann-Whitney, *p* = 3.20e^−20^). We also examined the correlation between co-occurence of m^6^A sites and the genomic distance between the set of m^6^A pairs analyzed, finding no positive correlation (Spearman’s *ρ* = −0.161) (Fig. 5F). Thus, our results suggest that m^6^A modifications preferentially co-occur in the same RNA molecules, independently of the distance between the two sites, implying that the deposition of an m^6^A modification in a given mRNA molecule is more likely to occur on mRNA molecules with a previous m^6^A modification.

### m^6^A stoichiometry does not largely vary across isoforms

We then wondered whether m^6^A modification stoichiometries of a given m^6^A site were similar or distinct across isoforms, as we could eyeball some genes that apparently showed different m^6^A modification stoichiometries across isoforms (Fig. 5G). To address this question systematically and in a transcriptome-wide fashion, we used high-coverage HepG2 human datasets (Additional File 2: Table S4). Firstly, we examined whether per-isoform m^6^A stoichiometries were replicable across independent biological replicates, finding an overall Spearman’s correlation of 0.83 between biological replicates sequenced in different flowcells (Fig. 5H, see also *Methods*). We then examined whether the modification stoichiometry of m^6^A sites varied consistently across isoforms. To this end, we analysed the variation in isoform-specific modification stoichiometry of m^6^A sites that showed a minimum per-isoform coverage of 40 reads in both replicates (*n* = 167 sites). Analysis of differential modification levels revealed only modest differences in m^6^A modification stoichiometries across isoforms (Fig. 5I), suggesting that m^6^A stoichiometry is in general not isoform-specific. Indeed, most of the identified m^6^A changes across isoforms were not replicable across biological replicates, suggesting that the initial variations observed across isoforms were caused by random sampling differences. The only site that we found to be significant was found in the EDF1 gene, which showed a replicable 20% stoichiometry difference between two isoforms (Additional File 1: Figure S13B). Notably, the lowest m^6^A frequency was found in the isoform that had a splicing site ~ 20nt downstream from the m^6^A-modified site.

Finally, we explored whether the variability of m^6^A frequency between isoforms would correlate with the distance between the m^6^A site and the exon junctions and/or polyadenylation sites, finding a modest negative correlation that was not significant (Spearman’s *ρ* = −0.12, *p* = 0.12, Additional File 1: Figure S14A). When splitting the sites into “varying” (mean frequency difference > = 5%) and non-varying sites, we observed that varying sites were slightly closer to the exon boundary than non-varying sites, but this difference was not statistically significant (Mann-Whitney-Wilcoxon test *p* = 0.6581).

### Applicability of NanoRMS2 method beyond m^6^A RNA modifications

As discussed earlier in this work, the per-read ‘labels’ used for training the *m*^*6*^*ABasecaller* were generated through predictions made by *NanoRMS2* on both wild type and METTL3 knockout samples, but this method is applicable to samples devoid of any modification of interest. Thus, the proposed method described in this work is only not applicable for training models that can predict m^6^A RNA modifications, but can be extended to any RNA or DNA modification of interest, using as input both native (modified) reads and unmodified reads.

To demonstrate the extended applicability of the method, here we employed NanoRMS2 to predict high-accuracy labels that can be in turn used to train a basecaller for predicting m^5^C and m^6^A DNA modifications. To this end, we ran NanoRMS2 on *E. coli* native (modified) and PCR-amplified (unmodified) DNA datasets. NanoRMS2 predicted modifications in 2 different sequence contexts: CCWGG (which is the motif for the m^5^C *Dcm* writer) and GATC (which is the motif for the m^6^A *Dam* writer) (Additional File 1: Figure S15A). Similarly, we then ran NanoRMS2 on *H. sapiens* NA12878 native and PCR-amplified DNA [86], in which NanoRMS2 identified as top-ranked ‘motif’ the CpG sequence context, which is the known motif for DNA m^5^C in eukaryotes, the most abundant DNA modification type (Additional File 1: Figure S15B). Finally, we ran NanoRMS2 on *S. cerevisiae* WT and ΔIme4 samples, which predicted GGACA context as the most frequent to be m^6^A-modified (as previously reported in literature for Ime4 [87]) (Additional File 1: Figure S15C), demonstrating that the GGACT-enriched motif of *m*^*6*^*ABasecaller*-predicted m^6^A sites in human and mouse is not inherent to a bias of the algorithm, but reflecting the natural biological variability in terms of preferences in motifs in one species or another one. Finally, we also examined whether METTL14 KO might show a different preference in terms of motifs compared to METTL3 knockout, finding that the captured motif by NanoRMS2 is largely GGACT-enriched, suggesting that METTL14 is not showing significant differences in terms of motif binding preferences compared to METTL3 (Additional File 1: Figure S15D), in agreement with previous works [19].

### Applicability to new RNA004 chemistries

In the past few months, RNA004 DRS chemistries have been made available, with promising preliminary results pointing to increased basecalling accuracies and sequencing yields, compared to previous RNA002 chemistries. The new chemistry does not only come with the deprecation of RNA002 chemistries, but also of key softwares and bioinformatic pipelines that were routinely used by the scientific community to analyse DRS datasets, including the basecalling algorithm *Guppy*, the resquiggling algorithms *Tombo* (https://github.com/nanoporetech/tombo) and *Nanopolish* (https://github.com/jts/nanopolish), the demultiplexing tool *DeePlexiCon* [88] and several NextFlow workflows for DRS data analysis, such as *MasterOfPores* (https://github.com/biocorecrg/master_of_pores) and *nf-core nanoseq* (https://github.com/nf-core/nanoseq). Notably, with the deprecation of these tools, all RNA modification detection tools developed to date have also became deprecated, as each of them relied on one or more of these softwares mentioned above.

With the release of the RNA004 chemistry, ONT made available several modification-aware base-caling models for the newer chemistry, similar to the *m6ABasecaller* presented in this work. While these models are highly promising, they are so far limited to very few RNA modifications (m^6^A, m^5^C, Ψ and inosine), they have not been peer-reviewed or benchmarked by the community in terms of false positives and/or false negatives, and their ability to detect modifications when other RNA modifications are nearby remains unexplored. Moreover, it is unlikely that ONT will release trained base-calling models for minority RNA modifications, such as those that are exclusively present in rRNAs, tRNAs or viral RNAs, or for non-natural modifications, such as those typically incorporated in RNA aptamers, despite being highly relevant to diverse research fields. Therefore, even with the appearance of ONT base-calling models, there is a great need to have alternative methods to detect RNA modifications in DRS datasets sequenced with RNA004 chemistries.

To address this gap, we re-established the *NanoRMS2* pipeline to make it compatible with the RNA004 chemistry. Key variations compared to our original pipeline include the use *remora* as a new resquiggling software (replacing *tombo,* https://github.com/nanoporetech/remora), and *bonito* as basecalling software (replacing *guppy,* https://github.com/nanoporetech/bonito). Of note, *dorado* was not chosen as basecalling software as it does not report ‘trace’ as feature, which we found was one of the top-performing features to differentiate modified and unmodified bases in our models, also in RNA004 chemistry (Additional File 1: Figure S16), as shown for RNA002 (Additional File 1: Figure S3).

To examine the accuracy of our adapted pipeline, we generated a new batch of ‘curlcake’ constructs [44], which were sequenced with the new RNA004 chemistry (Additional File 1: Figure S17). We reasoned that including both RNA modifications for which ONT modification-aware base-calling models are available (m^6^A, m^5^C and Ψ) and for which they are unavailable (ac^4^C, m^1^Ψ, hm^5^C and m^5^U) would maximise the use of the sequences by future researchers interested in retraining their own models and algorithms. Our results showed that this approach accurately distinguished modified from unmodified sequences in distinct 7-mers, for each of the 7 RNA modification types examined, with Receiver Operating Characteristic (ROC) Area Under the Curve (AUC) values ranging from 0.88 to 0.97 when using *fast* basecalling models, and 0.92 to 0.98 when using high accuracy (*hac*) basecalling models (Additional File 1: Figures S18 and S19). As a comparison, we examined whether this method would be applicable to older RNA002 chemistries, and whether the performance would be similar. Our results show that this pipeline yields similar performance in RNA002 chemistries, with ROC AUC values ranging from 0.87 to 0.98 for *hac* model, and 0.84 to 0.98 for *fast* model (Additional File 1: Figure S20). Overall, our work provides a promising proof-of-concept on the use of *remora* + *bonito* as alternatives to extract features (signal intensity, dwell time, trace) that can in turn be used to train novel RNA modification base-callers and updated RNA modification prediction algorithms compatible with latest RNA004 chemistries.

## Discussion

Nanopore sequencing technologies are revolutionising the fields of genomics and transcriptomics. Despite their potential to improve the precision, quality and complexity of existing DNA and RNA modification maps, long-read sequencing methodologies have still not been adopted as a mainstream sequencing technology. A major obstacle for their widespread adoption stems from the lack of modification-aware base-calling algorithms that will work for any given sequence context [89, 90].

To adequately study the role, function and dynamics of RNA modifications, technologies that can sequence RNA molecules in ways that preserve the native modifications are sorely needed, as well as algorithms that can identify them with single nucleotide and single molecule resolution. A major challenge to achieve this, however, has been to obtain high-quality and diverse ‘training sets’ for multiple modifications, and across diverse sequence contexts [89, 90]. Indeed, most RNA modifications cannot be synthesised chemically or enzymatically to provide such training standards. Thus, the generation of standards for all modifications is a major challenge in training nanopore or any other technology heavily relying on computational methods to map DNA or RNA modifications accurately [89].

In the last few years, several works have successfully shown that m^6^A RNA modifications –as well as other RNA modifications– can be detected using nanopore sequencing [39, 44–48, 51, 56, 58, 59, 65, 91, 92]. However, most methods developed so far often lack single molecule resolution (providing m^6^A predictions at per-site level), require computationally-intensive steps such as resquiggling, require the analysis of aggregated per-read information (so per-read predictions are not fully independent from other reads, and are affected by sequencing depth and per-site coverage), and/or have relatively high false positive and false negative rates [50, 93]. Here we address these limitations with the development of a modification-aware basecalling model, the *m*^*6*^*ABasecaller* (Fig. 1), which can produce m^6^A predictions in individual reads during the basecalling step, thus allowing us to address questions regarding the mechanism of m^6^A deposition in mRNAs at an unprecedented resolution, such as deciphering the interplay between m^6^A modifications and polyA tail lengths (Fig. 5A,B), learning the rules of m^6^A deposition within same reads (Fig. 5D-F) with regards to intron-exon junctions (Fig. 5C) and across isoforms (Fig. 5G).

Modification-aware basecalling models, such as the *m*^*6*^*ABasecaller*, show several key advantages compared to previous methods to detect modifications in DRS datasets, including those previously reported to achieve single-molecule resolution. Firstly, per-read predictions of m^6^A modified bases are fully independent from the rest of reads; by contrast, other single-read m^6^A detection methods require several reads to perform a statistical test that is needed for their final per-read predictions [38, 91]. Secondly, modifications are predicted de novo as the read is being sequenced, during the basecalling step, thus allowing potential coupling of m^6^A detection with other ‘live basecalling’ features, such as adaptive sampling’ [94–96] in the near future. Thirdly, they do not require a control sample (knockout or unmodified) to perform its predictions, nor a minimum sequencing coverage to predict a nucleotide as m^6^A-modified; the prediction is performed per-read and per-nucleotide, independently of other reads, m^6^A sites, or datasets. Fourthly, modifications are detected at the basecalling step, thus skipping all the heavy GPU/CPU computational efforts (resquiggling, feature extraction, statistical analyses, and/or additional post-processing) typically associated with the detection of m^6^A RNA modifications in nanopore DRS datasets [38, 39, 51, 65, 91]. Finally, the method will not be restricted to detecting RNA modifications in a specific subset of k-mers, nor requires prior knowledge of the motif; rather, it will predict RNA modifications in the biological contexts in which the RNA modification is naturally found, as these were the ones used to train the model.

A key feature of this work, in addition to the *m*^*6*^*ABasecaller *per se, is that it provides a novel approach for generating high-quality training sets that can be used to train models for other RNA modifications. Specifically, we propose a novel solution to generate training sets to train basecalling algorithms that consists in using biologically-derived data filtered to keep only high confidence modification labels (after multiple training rounds) to train the final basecalling model (Fig. 2). We show that these ‘high quality labels’ are improved when using features extracted from data that was previously basecalled with “modification-unaware” (IVT/PCR) models (Additional File 1: Figure S3). Once the basecalling model is trained using these ‘high-confidence’ labelled reads, sequencing data from any condition can be basecalled, and modifications will be predicted for each read and nucleotide. In this work we demonstrate the applicability of this approach, *NanoRMS2*, to generate high confidence labels to train an m^6^A-aware basecalling model, but the same approach is in principle applicable for any given RNA or DNA modification. To illustrate this, we demonstrate how our approach can be used to accurately label and train models to basecall bacterial m^5^C modifications (GGWCC contexts), bacterial m^6^A modifications (GATC contexts) and human m^5^C modifications (CpG contexts) (Additional File 1: Figure S15), demonstrating its potential use by future users to extended lists of RNA and/or DNA modifications.

To our surprise, we find that basecalling models trained with synthetic RNAs show poor performance in vivo (Additional File 1: Figure S1) this is true even if the synthetic molecules cover all possible 5-mers, such as in the case of the ‘curlcakes’ [44]. We hypothesise that the ‘chunks’ used by the neural network to make its predictions are longer than 5-mers, and therefore, the training data lacks sufficient sequence complexity. Consequently, these models suffer from two issues: i) they basecall poorly sequence contexts that were not present in the training set, and ii) they incorrectly predict reads ‘chunks’ to be fully modified or fully unmodified. One possible solution would be the use of synthetic molecules in which the modification of interest would be surrounded by randomised sequenced contexts,. However, we should note that these approaches will be limited by the chemical availability of the modification of interest in the form of phosphoramidite for solid-phase synthesis.

We should note that the *m*^*6*^*ABasecaller* was trained on human DRS datasets (see *Methods*). While we demonstrate that the *m*^*6*^*ABasecaller* performs well in other species such as mouse and zebrafish (Fig. 4 and Additional File 1: S4-5), its performance will not be optimal in species that have m^6^A modifications in very distinct sequence contexts than those present in human (DRACH). Indeed, the model will be unable to recognize m^6^A in sequence contexts that are not present in human, because it cannot predict m^6^A modifications in a sequence context that it had never seen during the training step. Thus, while *m*^*6*^*ABasecaller* will predict m^6^A modifications in any species, for species that are evolutionarily distant to human, we recommend generating a new m^6^A-aware basecalling model trained with data from that species of interest, following the same procedure as described here.

In this work, we reveal that m^6^A modifications are preferentially deposited in RNA molecules that contained a previous m^6^A modification, that m^6^A modification frequencies do not largely differ across isoforms, and that, contrary to the common belief, m^6^A-modified molecules hold longer polyA tails than their unmodified counterparts (Fig. 5), arguing against m^6^A-mediated mechanisms as main drivers of mRNA deadenylation and decay. Notably, the *m*^*6*^*ABasecaller* has also allowed us to identify unexpected issues that may arise when using inducible knockout (iKO) or knock-down (KD) systems as ‘control’ conditions. Indeed, we find that most m^6^A-modified sites are still present at detectable levels upon shorter induction times (Fig. 4E-F) suggesting that caution should be taken when using these systems and interpreting their results. Moreover, our work demonstrates that the loss of METTL3 protein in inducible systems is often not sufficient to prove that m^6^A is absent in mRNAs, and that, even if at lower stoichiometries, m^6^A modifications will still be present in certain RNA molecules.

While base-calling models for RNA004 chemistries are available, the *m6ABasecaller* offers the possibility to examine datasets generated with the previous chemistry, for which no alternative modification-aware basecaller exists. In addition, the feasibility to reimplement the approach for RNA004 chemistries enables future users to be able to build RNA and DNA basecalling models for modifications that might not be targeted by ONT, such as those that might not be of generalised interest. Thus, it is of pivotal importance to ensure that the community will be able to keep exploring the native RNA epitranscriptome using independent tools that can complement efforts done by ONT, as well as to assess potential biases of ONT-released modification-aware basecalling models.

## Conclusions

Our work provides a novel framework to generate high quality training sets and train modification-aware basecallers. We demonstrate that these tools enable transcriptome-wide RNA modification mapping with single-molecule and single-nucleotide resolution, with high specificity and high sensitivity, and retaining isoform information, opening its use for a variety of applications, including the direct detection of RNA modifications in clinical samples using nanopore sequencing.

## Methods

### Generation of PCR/IVT modification-unaware basecalling models

The ‘default’ basecalling models provided by ONT (such as those included in Guppy version 3.6.1, namely *dna_r9.4.1_450bps_hac.cfg* and *rna_r9.4.1_70bps_hac.cfg* for DNA and RNA basecalling, respectively) have been trained on wild-type (native) RNA and DNA molecules from human, yeast and *E. coli*. Consequently, all modifications that are naturally present in any of these species (such as m^6^A in DRACH context for RNA), will be trained into the model, and the model will learn to recognise them as canonical bases. To overcome this issue, we generated modification-unaware basecalling models (*dna_r9.4.1_450bps_pcr_hac.cfg* for DNA and *rna_r9.4.1_70bps_ivt_hac.cfg* for RNA), which were trained with unmodified DNA (PCR-amplified from *E.coli* whole genome*)* or unmodified RNA (in vitro transcribed from CEPH1463 cells, UCSC_Run1_IVT_RNA) datasets using a customised version of *taiyaki*. Because highly expressed transcripts are known to typically dominate cDNA/RNA sequencing experiments, we balanced the RNA training sets taking up to 5 reads per each transcript. Modification-unaware trained models are available in *NanoRMS2* GitHub repository (https://github.com/novoalab/nanoRMS2/). All models trained as part of this work are listed in Additional File 2: Table S1.

### Obtaining high-confidence per-read labels with NanoRMS2

*NanoRMS2* is a software that can generate high-confidence labelled data from biological samples, which can in turn be used to train modification-aware basecalling models. that will be highly accurate in predicting RNA modifications in in vivo contexts. The NanoRMS2 pipeline takes as input a reference sequence and two direct DNA or RNA sequencing samples. The first sample is expected to have little-to-no modification, while the second should have at least some base modifications present in biologically relevant sequence contexts. The first sample can be generated by amplification (PCR) for DNA or in vitro transcription (IVT) for RNA. Alternatively, this sample can be obtained from knock-out (KO) or knock-down (KD) of specific modification writer enzymes.

Briefly, *NanoRMS2* performs following steps (Fig. 2): i) extraction of basecalling features for all bases in all reads: signal intensity (SI), Modification Probability (MP), Dwell-Time at the position 0 (DT) and 10 bases upstream (DT10), and Trace values for the reference nucleotide (TR) and all canonical bases (TA, TC, TG and TT, for A, C, G and T/U, respectively; ii) data aggregation for all 7-mers from entire genome/transcriptome, iii) pre-selection of candidate modified kmers using Kolmogorov–Smirnov test, iv) combination of semi- and supervised learning to differentiate between un- and modified reads at pre-selected positions, v) motif enrichment analysis to further limit the analysis only to the sequence motifs that correspond to modification writers, vi) encoding of the high confidence labels into BAM files. The labels generated by NanoRMS2 will then be used to train the final modification-aware basecalling model (see next section).

### Generation of an m6A-aware basecalling model

The modification-aware (m^6^A) basecalling model benchmarked in this work (*m*^*6*^*ABasecaller*) was trained as follows: first, we trained a modification-unaware model with *taiyaki* using only unmodified (IVT) reads, which were taken from UCSC_Run1_IVT_RNA dataset, which is publicly available from the Nanopore WGS consortium [63]. This model was then used to base-call Human WT rep1 and METTL3 KO rep1 reads from PRJEB40872, which were then used by *NanoRMS2* to label reads present at m^6^A sites as modified or unmodified [45]. Finally, high-confidence, per-read modification predictions were used as ‘labels’ to train the m^6^A basecalling model using *taiyaki*. We used at most 2 modified and 2 unmodified reads for every position of every transcript. The final trained m^6^A-modification aware basecalling model (*rna_r9.4.1_70bps_m6A_hac.cfg*), which we refer to as ‘*m*^*6*^*ABasecaller*’, is available in *GitHub* (https://github.com/novoalab/m6ABasecaller), including detailed description and examples on how to use this model and how to process the output files generated by the *m*^*6*^*ABasecaller*. We should note that the *m*^*6*^*ABasecaller* (*rna_r9.4.1_70bps_m6A_hac.cfg)* has a slightly lower accuracy (88.9% identity to reference) compared to the default RNA model (91.1% identity to reference), which we attribute to the basecalling of 5 letters instead of 4.

### mESC culturing and passaging

*M. musculus* embryonic stem cells (mESC E14tg2A) were cultured under feeder-free conditions using 0.1% Gelatine (Millipore, #ES-006-B) coated plates (ThermoFisher, #140675) and grown in mESC medium prepared as follows: KnockOut™ DMEM (ThermoFisher, 10829018) supplemented with 10% FBS (in-house tested for ES competence) MEM Non-Essential Amino Acids Solution 1X (ThermoFisher, #11140050), GlutaMAX™ 2 mM (ThermoFisher, #35050061), Pen/Strep 1X (ThermoFisher, #15140122), β-mercaptoethanol 50 µM (Gibco, #31350010), HEPES 30 mM (Gibco, #15630080), 0,22 µm filtered and then supplemented with LIF conditioned (may 2021- in house tested). Cells were passed every 2-3 days in a 1:6-1:8 dilution.

### Generation of tamoxifen-inducible METTL3 knockout mES cells

In order to obtain tamoxifen-inducible METTL3 Knock-Out mES cells, 10^6 E14 mES (mESC E14tg2A) cells were transformed via electroporation (EP) with 3 ug of Piggybac MerCreMer Addgene plasmid (#124,183) and 9ug pBase plasmid (PL623—Wellcome Trust Sanger Institute) in NEPA21 electroporator. Electroporated (EP) cells were then selected with 400 ug/mL Neomycin (Roche, #04727878001) and resistant clones (mES cell line expressing Cre) were established. Subsequently, cells carrying the MerCreMer system were used for the insertion of 2 LoxP sites flanking a fragment of METTL3 gene. Briefly, the LoxP1 site was inserted by electroporation of 12,2 uM RNP Cas9 protein (PNA BIO, #CP02), 16 uM sgRNA1 and 4,8 uM ssODN (LoxP1 sequence) in OptiMEM (Gibco, #11,058–021). 24 h post-EP the cells were sorted, seeded at single cell and genotyped by PCR and Sanger sequencing. One positive clone was then selected for the insertion of the LoxP2 sequence using the same procedure as described above with sgRNA2. 48 h post-EP, the cells were sorted, seeded at single cell and genotyped by PCR and Sanger sequencing. All sequences used to generate this cell line can be found in Additional File 2: Table S14, and were taken from Wang et al., 2018 [79].

### Treatment of mESC with STM2457 inhibitor and with tamoxifen

The METTL3 inhibitor STM2457 (STORM Therapeutics) was administered to mESC cultures to a final concentration of 2, 10 or 20 µM. Dilutions of STM2457 were performed in DMSO (PanReac AppliChem, #A3672). As a control, the same volume of DMSO was administered to the cells. Cells were harvested with Trizol (Invitrogen,15596018) 24 h after the addition of the inhibitor. For tamoxifen treatment, (Z)−4-Hydroxytamoxifen (Sigma, #H7904-5MG) (diluted in MetOH) was added to the culturing media to a final concentration of 2.5 µM at each medium replacement until harvest day. As a control, we added the same volume of vehicle (MetOH). Cells were harvested in Trizol (Invitrogen, #15596018) 6 or 14 days after tamoxifen addition to the media.

### Western blotting

To perform the Western Blot assay, cells were lysed in RIPA buffer containing protease inhibitor (Roche, #11873580001) and lysates were centrifuged at 15000 rcf for 15 min at 4 °C to remove cellular debris. Lysates were then prepared with NuPAGE sample buffer LDS 4X (Thermo Fisher, #NP0007) and NuPAGE Sample Reducing Agent (Thermo Fisher, #NP0004), and were loaded on 15% acrylamide gels. Protein content was previously checked with Ponceau (Sigma-Aldrich, #P7170) and membranes (Merck, #IPVH00010) were blocked with TBS-T BSA 3%. METTL3 antibody (Abcam ab195352) was used at a 1:1000 dilution, and GAPDH antibody (Abcam ab8245) was used at 1:10.000. Anti-mouse (Agilent #P0260) or anti-rabbit (Abcam, #ab6721) HRP-conjugated secondary antibodies were diluted 1:5000 in TBS-T.

### Total RNA extraction

For each condition, 3 wells of a 6-well plate of mESC (corresponding to 6*10^6 cells approximately) were resuspended in 1 mL Trizol (Invitrogen,15596018) vortexed two times for 30 s and incubated 5 min at room temperature, then processed immediately or stored at −80. Next, 200 µL of chloroform (Sigma, C2432) was added to each sample, mixed for 20 s, incubated for 2–3 min at room temperature and centrifuged at 16,000 g for 15 min at 4ºC. The resulting upper aqueous phase was transferred to a new tube and mixed with 500 µL of molecular grade 2-propanol (Sigma, I9516). Samples were incubated 10 min at room temperature and centrifuged at 12,000 g for 10 min at 4ºC to precipitate the RNA. The pellet was washed with 70% ethanol and centrifuged at 7,500 g for 5 min at 4ºC, air-dried for 10 min and eluted in nuclease free water.

### PolyA selection from mESC total RNA

Total RNA was DNAse-treated (Ambion, AM2239) at 37ºC for 20 min, and cleaned up using RNeasy MinElute Cleanup Kit (Qiagen, 74,204). 70–100 ug of total RNA was then subjected to double polyA-selection using Dynabeads Oligo(dT)25 (Invitrogen, 61,002) following manufacturer’s protocol and eluted in ice-cold 10 mM Tris pH 7.5.

### Generation of in vitro transcribed ‘curlcake’ sequences

This study used synthetic RNAs known as ‘curlcakes’, which were designed to include all possible 5-mer sequences while minimising RNA secondary structures [44–51], and consist of four *in-vitro* transcribed constructs: Curlcake 1, 2,244 bp; Curlcake 2, 2,459 bp; Curlcake 3, 2,595 bp and Curlcake 4, 2,709 bp. To generate ‘curlcake’ synthetic RNAs, the plasmids were digested with BamHI-HF (NEB, R3136L) and EcoRV-HF (NEB, R3195L) followed by a clean-up using phenol/chloroform/isoamyl-alcohol 25/24/1, v/v, pH = 8.05 (Sigma Aldrich, P3803). Linearized plasmids were used for in vitro transcription with the AmpliScribe T7-Flash Transcription Kit (Lucigen, ASF3507) adding unmodified rATP and N6-methyladenosine triphosphate (m6ATP, TriLink Biotechnologies, N-1013–5), N5-methylcytosine triphosphate (m5CTP, Trilink, N-1014–1), 5-hydroxymethylcytosine triphosphate (hm5CTP, Trilink, N-1087), N4-acetylcytosine triphosphate (N4-Acetyl-CTP, Jena Bioscience, NU-988S), N1-methylpseudouridine triphosphate (m1meΨTP, Jena Bioscience, NU-890S), pseudouridine triphosphate (ΨTP, Trilink, N-1019–1) and N5-methyluridine triphosphate (m^5^UTP, Trilink, N-1024–1). All constructs were polyadenylated using Escherichia coli poly(A) polymerase (NEB, M0276S) according to manufacturer’s instructions and purified with RNAClean XP beads. The addition of poly(A) tail was confirmed using Agilent 4200 TapeStation and the concentration was determined using Qubit Fluorometric Quantitation.

### Direct RNA nanopore library preparation and sequencing

#### For RNA002

PolyA( +) selected (for in vivo samples) or in vitro polyadenylated (for synthetic *curlcakes*) RNA samples were prepared for nanopore sequencing using the direct RNA sequencing (DRS) kit (SQK-RNA002), following the ONT protocol version DRS_9080_v2_revI_14Aug2019 with half reaction for each library until the RNA Adapter (RMX) ligation step, with some adaptations. Briefly, 250 ng of polyA( +) RNA were ligated to pre-annealed custom RT adaptors (IDT) containing barcodes [88] with T4 DNA concentrated Ligase (NEB-M0202M) for 15 min at RT. Next, reverse transcription was performed during 15 min at 50ºC using SuperScript IV RT Enzyme (Invitrogen,18,090,050), followed by heat inactivation for 5 min at 70ºC. Ligated products were then purified using 1.8X Agencourt RNAClean XP beads (Fisher Scientific, NC0068576) and washed with 70% freshly prepared ethanol. For the last ligation step, 50 ng of reverse transcribed RNA from each reaction was pooled, mixed with RMX adapter, composed of sequencing adapters with motor protein, and incubated for 15 min in the presence of concentrated T4 DNA Ligase (NEB-M0202M). Finally the ligated RNA:DNA hybrid was purified using 1X Agencourt RNAClean XP beads, washed with Wash Buffer (WSB) twice. The sample was then eluted in Elution Buffer (EB) and mixed with RNA Running Buffer (RRB) before loading onto a primed R9.4.1 flowcell, and ran on a MinION sequencer.

#### For RNA004

For RNA004 runs (Table S2), DRS library preparation was carried out according to the manufacturer’s instructions (direct-rna-sequencing-sqk-rna004-DRS_9195_v4_revB_20Sep2023-promethion). 100 ng of polyA( +) RNA were ligated to pre-annealed custom RT adaptors (IDT) containing barcodes [88] with T4 DNA concentrated Ligase (NEB-M0202M) for 15 min at RT. Next, reverse transcription was performed during 15 min at 50ºC using SuperScript IV RT Enzyme (Invitrogen,18,090,050), followed by heat inactivation for 5 min at 70ºC. Ligated products were then purified using 1X Agencourt RNAClean XP beads (Fisher Scientific, NC0068576) and washed with 70% freshly prepared ethanol. After elution, all reverse transcribed samples were pooled in one tube, and 500 ng of total cDNA were mixed with 6 µl of RNA Ligation Adapter (RLA), followed by 15 min incubation in the presence of concentrated T4 DNA Ligase (NEB-M0202M). Finally the ligated RNA:DNA hybrid was purified using 0.6X Agencourt RNAClean XP beads, washed with Wash Buffer (WSB) twice. Before loading the libraries, these were mixed with 100 µl of Sequencing Buffer (SB) and 68 µl of Library Solution (LIS). Libraries were run on a primed FLO-PRO004RA flowcell using a PromethION 2 Solo sequencing device.

### Basecalling, demultiplexing direct RNA sequencing data

Raw Fast5 files from dRNA sequencing runs analysed in this study (listed in Additional File 2: Table S2) were processed with Master of Pores pipeline version 3 [61], which is publicly available in GitHub (https://github.com/biocorecrg/MOP3). The *mop_preprocess* module was used to process the samples, using *DeePlexiCon* with default parameters [88] to demultiplex the runs when required, and basecalled with *Guppy* basecaller 3.4.5 (https://nanoporetech.com) using the m^6^A basecalling model trained as part of this work (*rna_r9.4.1_70bps_m6A_hac.cfg*, which is available at https://github.com/novoalab/m6ABasecaller/tree/main/basecalling_model).

### Extraction of modification information from DRS datasets basecalled with *m*^*6*^*ABasecaller*

m^6^A-basecalled Fast5 files were processed with ModPhred [62] (https://modphred.readthedocs.io/en/latest/) to encode the modification probabilities (calculated by the *m*^*6*^*ABasecaller*) into Fastq files. ModPhred was also used to map the Fastq files with minimap2 [97] (version 2.17-r941) with “*-ax map-ont -k13”* parameters to the hg38 genome for human data, mm10 genome for mouse data, and GRCz11 for zebrafish data. ModPhred was also used to generate a compressed bedMethyl (*.mod.gz) file with a list of m^6^A sites, which were considered as those positions with coverage > = 25 and modification frequency > = 5%, using as modification probability 0.5.

The output file generated by ModPhred (*mod.gz*) was then processed to identify replicable m^6^A sites using a custom python script. Replicable m^6^A sites were defined as those sites with coverage > = 25 and their respective modification frequency > = 5% in all replicates. Metagene plots depicting the distribution of m^6^A sites were generated using the Guitar package version 2.14 [98]. As input we used a isoform annotation that was filtered to contain only those transcripts that were expressed in our samples, in order to avoid the presence of artificial peaks (as the package by default uses the mean 5’-UTR, CDS and 3’-UTR length of all annotated isoforms) for plotting the m^6^A distribution.

Motif enrichment analysis of the predicted m^6^A sites was performed using MEME version 4.11.2 [99] with the following parameters: *-nostatus -dna -mod zoops -nmotifs 5 -minw 2 -maxw 10*. To build m^6^A frequency scatter plots and density plots with logarithmic axis, a pseudocount of 0.001 was added to each value to allow for logarithmic transformation.The set of m^6^A sites predicted in human, mouse and zebrafish DRS datasets using *m*^*6*^*ABasecaller* are listed in Additional File 2: Table S5 (human HEK293T), Table S7 (mouse ES cells), Table S8 (zebrafish embryos 4hpf) and Table S13 (human HepG2).

### Isoform annotation

Per-read isoform annotation was performed on uniquely mapping reads, which were extracted using the samtools flag -F 3844, using Isoquant v2.2.2 [100] with “–stranded forward –complete_genedb –count_exons –data_type nanopore” parameters and the Ensembl annotation version 109. Isoquant was used to perform isoform predictions, and only those reads with “unique”, “fsm” (full splice match) or “mono_exon_match” tags were kept for downstream analyses. We should note that this filtering discarded ~ 80% of the mapped reads that could not be unambiguously assigned to a specific isoform. We realised that Isoquant annotations correctly assigned isoforms based on exon-intron junctions, but sometimes contained reads that corresponded to two distinct isoforms, which had either different 5’utr start sites or different 3’utr ends.

### PolyA tail length estimation

Per-read polyA tail length estimation was performed using the *mop_tail* module of MOP2, using *tailfindr* version 1.3 [101]. Per-read polyA tail length estimations were merged with per-read modification data and per-read isoform assignments, generating a final table that contained all features for every read.

### Analysis of m^6^A modification co-occurrence

To assess whether m^6^A modifications tend to occur in the same reads, we first filtered the DRS dataset to keep only full-length reads that were uniquely assigned to a given isoform. Then, we analysed the per-read m^6^A modifications for each pair of predicted m^6^A sites that met the following criteria: (i) both m^6^A sites were found in a transcript with coverage > = 200 reads, and (ii) the expected number of reads that contain two modified sites > = 2. If these criteria were met, the expected m^6^A co-occurrence of site A and B –quantified as ‘reads’ or ‘counts’– was calculated as follows:

$$Exp(A,B)= mod freq (A)* mod freq (B) * transcript coverage$$

Then, for each pair of m^6^A sites analysed, the number of standard deviations (NSD) from the expected counts was computed as follows:

$$NSD(A,B) =\frac{Obs(A,B) - Exp(A,B)}{\sqrt{Exp(A,B) * (1-(}mod freq (A) * mod freq (B))}$$

### Comparison of *m*^*6*^*ABasecaller* predicted m^6^A sites with orthogonal datasets

To compare the list of m^6^A sites predicted by the *m*^*6*^*ABasecaller* to those previously published and had been annotated under hg19 assembly, we lifted the genomic coordinates using the UCSC “Lift Genome Annotations” online tool (https://genome.ucsc.edu/cgi-bin/hgLiftOver) to hg38. To subset the bed files based on coverage (only sites with at least 25 reads coverage were included in our analysis), we used bedtools coverage tool (bedtools version v2.30.0). The m^6^A sites identified by *m*^*6*^*ABasecaller* as well as by other orthogonal studies (before and after filtering) are listed in Additional File 2: Table S10, and can be found in *m*^*6*^*ABasecaller* Github repository (https://github.com/novoalab/m6ABasecaller).

### Comparison of *m6ABasecaller *and m6Anet accuracy in curlcake mixtures

We generated subsets of 4000 reads from 0 and 100% m^6^A modified curlcakes (rep2) with 6,25%, 12.5%, 25% and 50% m^6^A modified reads using an in-house script, available here: https://github.com/novoalab/m6ABasecaller/blob/main/notebooks/m6Anet.ipynb. Briefly, we selected 1000 uniquely mapping, full-length reads per curlcake sequence (total of 4 ‘curlcakes’). Only full length reads were kept to ensure equal coverage of all positions. The list of read IDs used for these subsets is available in the *m*^*6*^*ABasecaller* GitHub repository at https://github.com/novoalab/m6ABasecaller_dev/tree/main/curlcakes_mixtures. The same input reads were used to benchmark *m*^*6*^*ABasecaller* and m6Anet. For m6Anet, we used default parameters according to the instructions of the repository (github.com/GoekeLab/m6anet) with the default model. Following the m6Anet recommended thresholds, we considered a site to be m^6^A-modified modification probability threshold, reported in the data.site_proba.csv output, was greater than 0.9.

### Training classifiers for RNA004 chemistry

For reads sequenced with RNA004 chemistry, 13 features from positions -1, 0 (modified base) and + 1 were extracted (39 features in total). These features originated from: i) signal intensity (SI) mean and standard deviation, ii) dwell time at a pore (DT0) and at a helicase (shifted by 10 bases, DT10), iii) basecaller features, i.e., probability of an A, C, G, T/U, N (stay signal) and a reference base; and iv) the three most likely k-mers returned by a CTC-CRF basecalling model. Signal-based features were retrieved using remora v2.1.3, whereas basecaller-based features were obtained using bonito v0.8.1. The features were extracted using two basecalling models: rna004_130bps_fast@v5.1.0 (fast) and rna004_130bps_hac@v5.1.0 (hac). Basecalled reads were aligned with minimap2 v2.28 using the following parameters: -k13 -w4 -n1 -m15 -s30 -A2 -B1 -O1,32 -E1,0.

Sequencing runs of synthetic modified and unmodified ‘curlcakes’ were downsampled to 1,000 reads per reference per sample, selecting the reads with the longest alignments. Features were then retrieved from each dataset as described above, and stored in BAM files as tags. Only positions centred at the modified base and containing only single modification within a 7-mer were used. For example, for m^6^A only BBBABBB positions were used. Subsequently, a classifier was trained for every selected 7-mer and for every sample (modification type). Reads originating from unmodified and modified samples were balanced to match the coverage of the sample with lower number of reads for a given position. Positions with fewer than 100 aligned reads were skipped. Data was split 50:50 into a training and testing set. Random Forest classifier was trained using a training set and evaluated on a testing set. Random Forest implementation from scikit-learn v1.5.2 was used. Receiver operating characteristic (ROC) curve and the total area under the curve (AUC) were calculated using scikit-learn v1.5.2.

## Supplementary Information

Additional file 1. Contains all the supplementary Figures for this manuscript.

Additional file 2. Contains all the supplementary Tables for this manuscript.

## Data Availability

Basecalled FAST5 generated as part of this work have been deposited in ENA under the accession numbers PRJEB61874 and PRJEB82528. The deposited data includes: (i) DRS RNA002 runs from in vitro transcribed curlcakes (12.5%, 25%, 50% and 75% of m6A), (ii) DRS RNA002 runs from mES cells treated with STM2457 (CTR, 2uM, 10uM and 20 uM); and (iii) DRS RNA002 runs untreated and tamoxifen-treated (6 days or 14 days) mES cells, where the tamoxifen treatment induces METTL3 KO; and (iv) DRS RNA004 runs from IVT curlcake constructs with different modifications. All other DRS datasets used in this work were taken from publicly available datasets [102–107], and are described in detail in Additional File 2: Table S2. The list of m6A-modified sites in HEK293T cells predicted by Illumina-based methods (GLORI-seq and m6ACEseq) used for orthogonal validation were taken from publicly available resources (GLORI-seq from Supplementary Data 1 of [29] and m6ACEseq from m6ACE-Seq.csv from [108]). The list of predicted m6A-modified sites in HEK293T cells predicted using miCLIP was kindly provided by Samie Jaffrey. Human NA12878 native and PCR-amplified DNA was taken from PRJEB30620 [86]. Code to generate high confidence datasets to train RNA basecalling models can be found in the NanoRMS2 GitHub repository (https://github.com/novoalab/nanoRMS2/) [109] and in Zenodo (10.5281/zenodo.14810895) [110]. The trained m6ABasecaller model is publicly available in the m6ABasecaller GitHub repository (https://github.com/novoalab/m6ABasecaller) [111] and in Zenodo (https://zenodo.org/records/14214259) [112]. De novo m6A-aware basecalling using the m6ABasecaller, as well as modPhred code required for downstream m6A modifications analyses, consisting of processing m6A-aware basecalled reads into per-site and per-read processed files, can be directly executed through the MasterOfPores [61] NextFlow workflow (version 3), which has been made publicly available at: https://github.com/biocorecrg/MoP3.
