# Supplementary material for: De novo basecalling of RNA modifications at single molecule and nucleotide resolution
Source: Genome Biol. 2025 Feb 25;26:38. doi: 10.1186/s13059-025-03498-6 (PMC11853310; doi:10.1186/s13059-025-03498-6)

**ADDITIONAL FILE 1**

**Supplementary Figures**

***De novo* basecalling of RNA modifications**

**at single molecule and single nucleotide resolution**

Sonia Cruciani^1,2,^*, Anna Delgado-Tejedor^1,2,^*, Leszek P. Pryszcz^1,^*^,#^, Rebeca Medina^1^, Laia Llovera^1^ and Eva Maria Novoa^1,2,3,#^

*^1^Centre for Genomic Regulation (CRG), The Barcelona Institute of Science and Technology, Dr. Aiguader 88, Barcelona 08003, Spain*

*^2^Universitat Pompeu Fabra (UPF), Barcelona, Spain*

*^3^ICREA, Pg. Lluís Companys 23, Barcelona, España*

* These authors contributed equally

^#^ Correspondence to: Leszek P. Pryszcz ([lpryszcz@crg.eu](mailto:lpryszcz@crg.eu)) and Eva Maria Novoa (eva.novoa@crg.eu)

**Figure S1. Benchmarking of base-calling models trained with m^6^A-modified and unmodified curlcakes and/or in vivo data supplemented with curlcakes. (A)** IGV snapshots of curlcake IVT constructs generated with different % of m^6^ATP as input (100%, 75%, 50%, 25%, 12.5% and 0%, from top to bottom). Reads were basecalled with the m^6^A model trained with curlcakes only. Predicted (observed) m^6^A modification frequency per-site is calculated as the sum of reads at a given site with modification probability greater than 0.5, and is shown next to each panel. Individual reads are shown as “squished” and bases are colored by their modification probability (see Legend). **(B)** IGV snapshots of reads in IME4 KO and WT yeast reads, basecalled with m^6^A model trained with ‘curlcakes’ and yeast data (see **Table S1** for detailed list of trained base-calling models). Individual reads are shown as “squished” and bases are colored by their modification probability (see Legend).

**
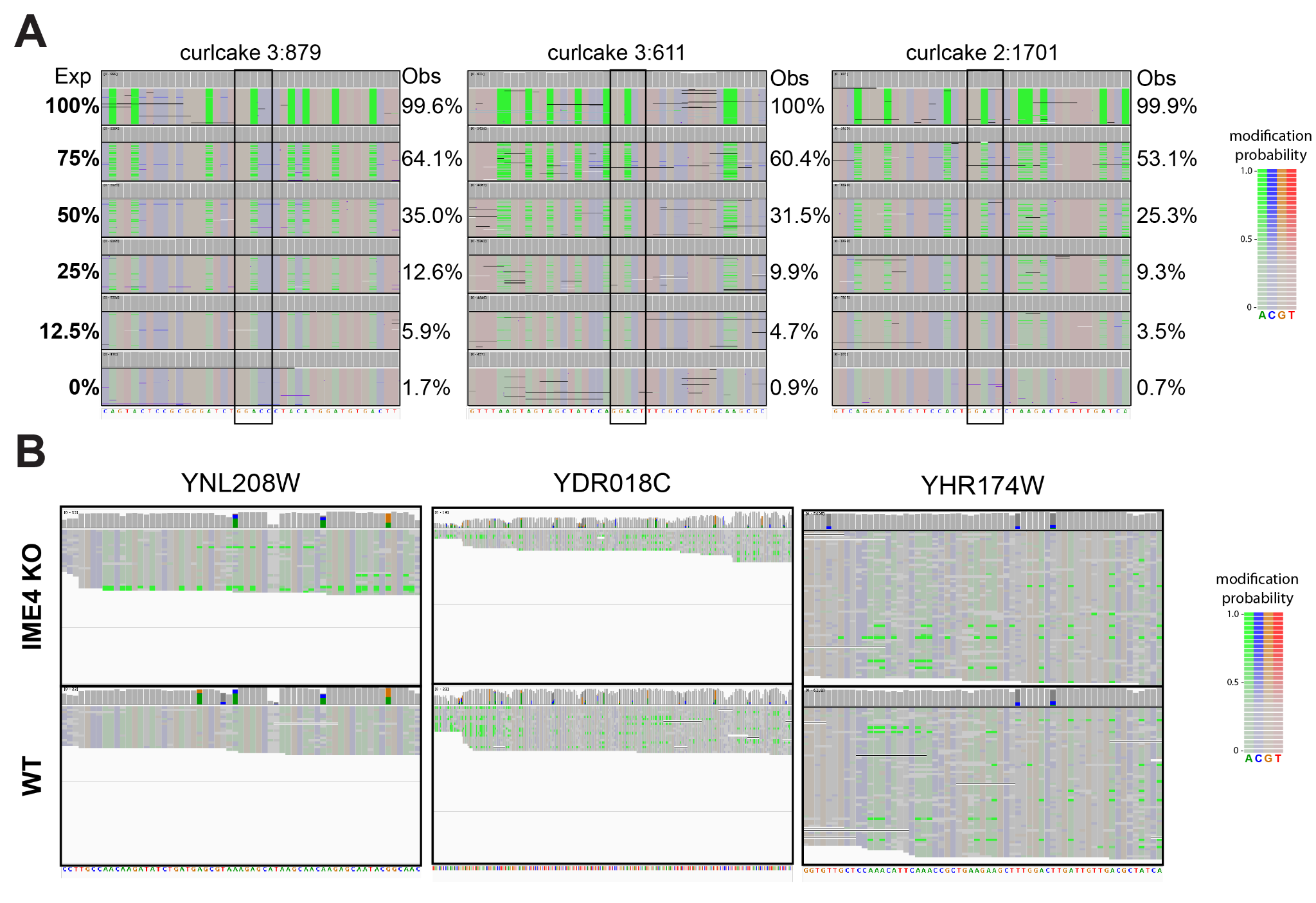
**

**Figure S2. Types of training data to train a modification-aware RNA or DNA basecalling model.** Current approaches used to obtain training datasets to train modification-aware basecalling models are depicted in panels A, B and C, illustrating their respective strengths and limitations. **(A)** Biologically derived datasets cover entire sequence complexity (suitable to train a basecaller), but lack modification information (we know for example that some reads in the WT are modified, but we don't know which ones). **(B)** Synthetic datasets provide ground truth (known modification status), but are not well suited to train a basecalling model due to the poor sequence complexity. **(C)** In this work, we propose the use of biological RNA reads coupled to in silico labeling to obtain a high confidence set of unmodified or modified reads to train the base-calling model. This approach provides biological complexity and per-position information about modification status for every read, and therefore it is well-suited to train a modification-aware basecalling model. The per-read labeling and per-site labeling is performed using a new tool developed as part of this work, *NanoRMS2*.

**
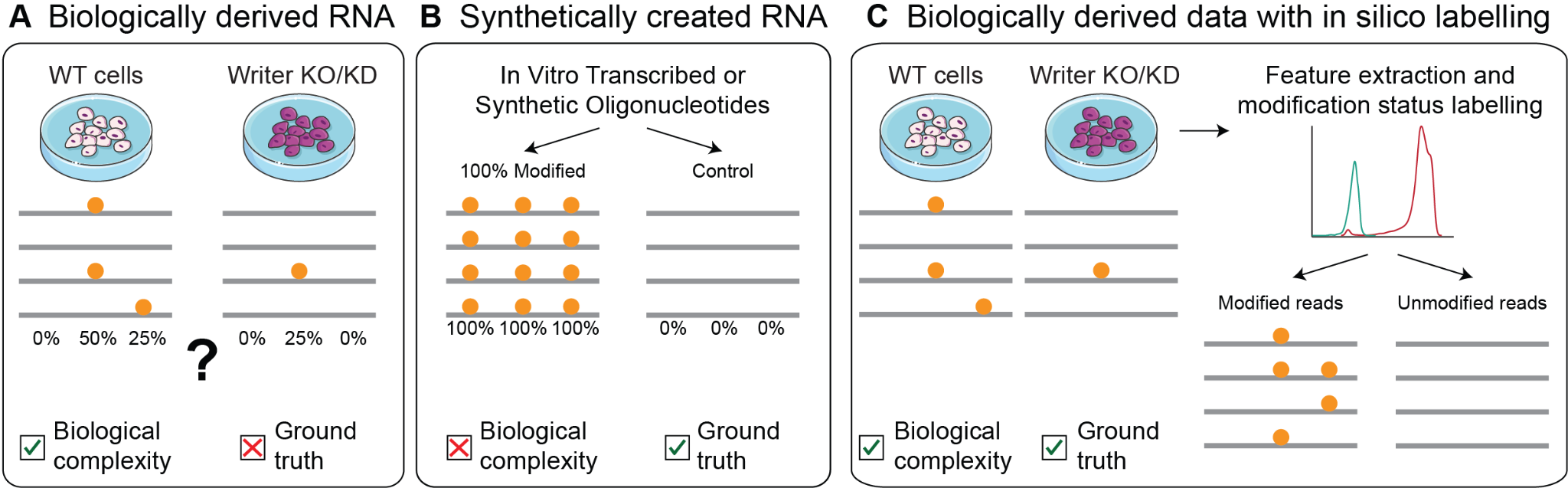
Figure S3. Comparison of signal intensity and trace features in modified and unmodified reads when using ‘default’ ONT basecalling model or modification-unaware PCR/IVT-trained basecalling model. (A,B)** Density plots of the Trace (TR) values observed in unmodified (blue) and modified (orange) k-mers, at positions -1, 0 and 1. TR values were obtained using a default base-calling model (upper panels) or the IVT/PCR-trained model (lower panels). We find that the difference in TR between modified and unmodified k-mers is maximized in IVT/PCR-trained models. **(C,D)** Boxplots depicting difference in signal intensity (C) and trace (D) between modified (native) and unmodified (whole genome amplified) DNA reads, when using default basecalling model (blue) or in-house trained canonical basecalling model (orange), which was trained only on unmodified DNA reads (PCR-amplified). Boxplots are shown for all 5 nucleotides of the 5-mer, centered at the modified position (0). K-mers have been chosen as they are expected to contain a modification for some of their reads, and hence a difference between native and unmodified DNA reads. **(E,F)** Boxplots depicting difference in signal intensity (E) and trace (F) between m^6^A-modified and unmodified RNA reads, when using default basecalling model (blue) or in-house trained canonical basecalling model (orange) that was trained only on unmodified (in vitro transcribed) reads. Boxplots are shown for all 5 nucleosides of the 5-mer, centered in the modified position (0). The Y axis represents the absolute difference between modified and unmodified samples for a given feature. The number of k-mers included in each boxplot is shown in the title (N=X). P-values were calculated using two-sided Mann-Whitney-Wilcoxon test and reported as either: ns (not significant), * (p <=0.05), ** (p <= 0.01), *** (p <= 0.001) or **** (p <= 0.0001).


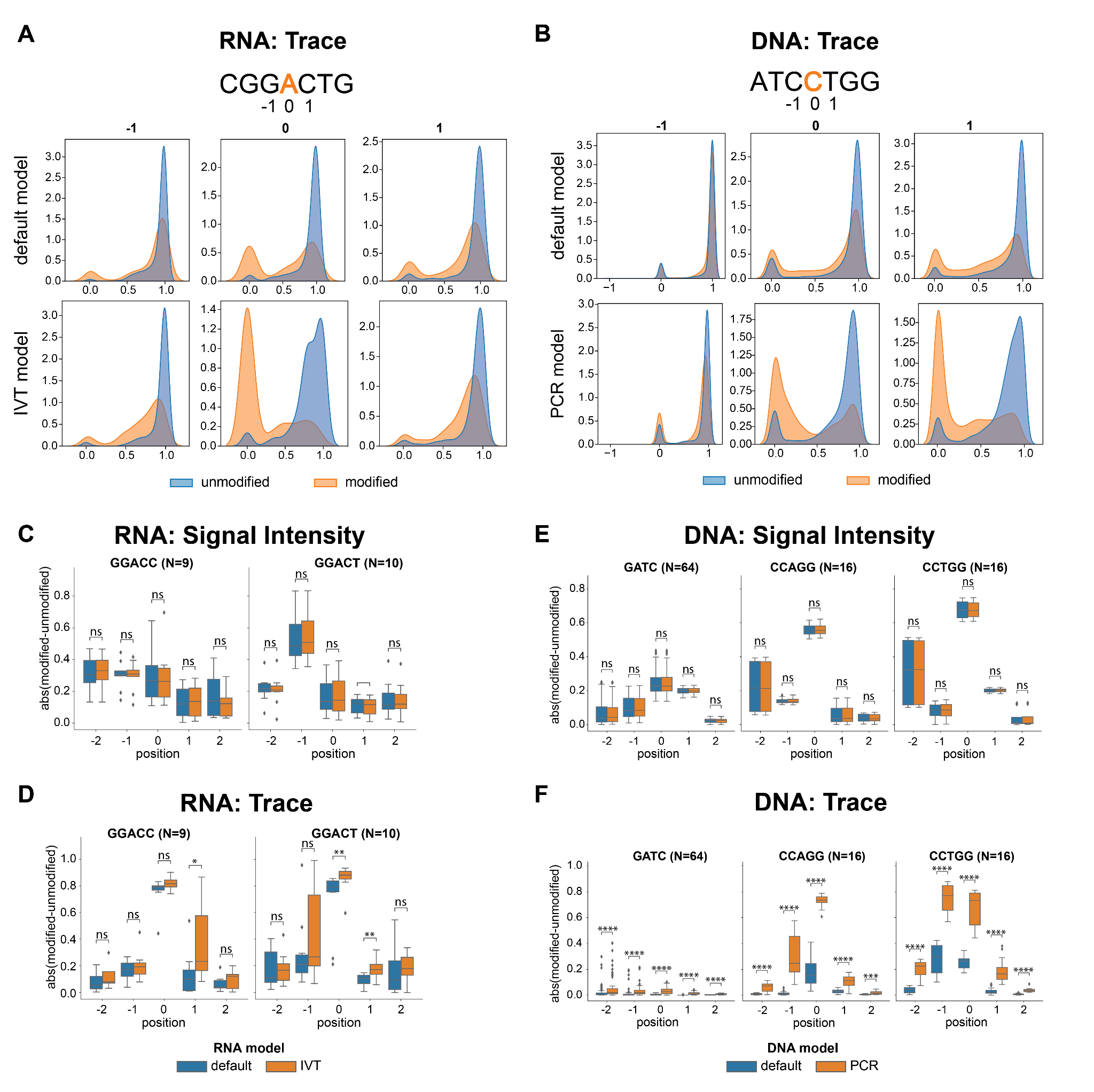


**Figure S4. Identification of optimal m^6^A modification probability threshold. (A)** Distribution of the modification probability (x axis) for each A in GGACT positions (N=14) covered by curlcake reads (N=1,336 sites in total for each type), for unmodified and m^6^A modified curlcake reads. Vertical dashed lines show the different modification probability thresholds of 0.01, 0.1 and 0.5. **(B)** Distribution of the modification probability (x axis) for each A in GGACT positions (N=2,248) covered by in vitro transcriptome reads (N=141,992 sites in total for each replicate), for two replicates of in vitro transcribed RNA (unmodified) from HeLa cells. Vertical dashed lines show the different modification probability thresholds of 0.01, 0.1 and 0.5. **(C)** ROC curve showing optimal (based on Youden Index) ratio TPR/FPR at modProb>=0.1 for ‘curlcake’ sequences (N=742 sites in total for 14 GGACT positions). **(D)** Boxplots of observed per-site m^6^A frequencies obtained from 0%, 25%, 50% and 100% modified curlcakes calculated using the 0.5, (left) 0.1 (middle) or 0.01 /(right) modification probability thresholds.

**
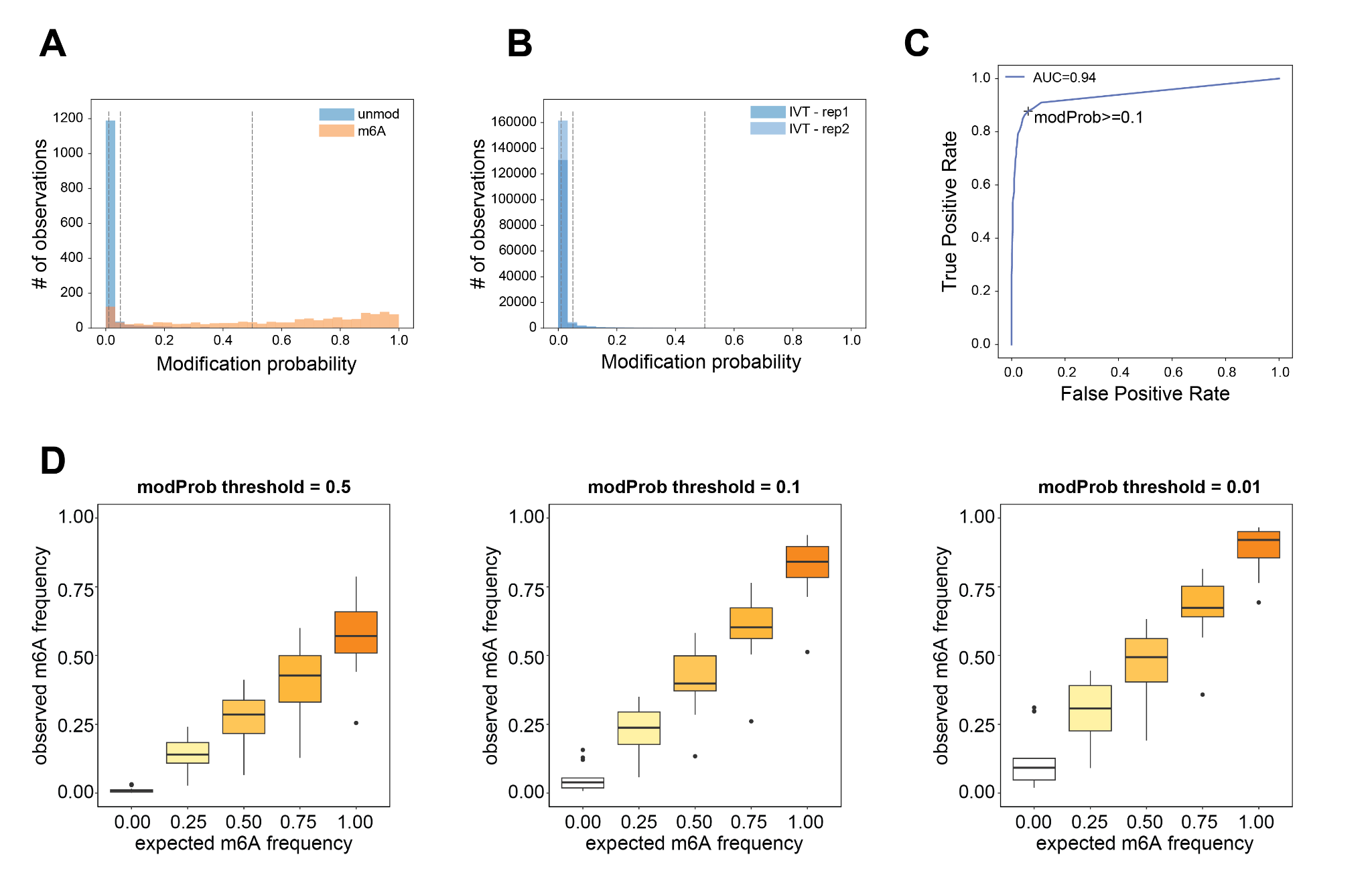
**

**Figure S5. Performance of *m^6^ABasecaller* in synthetic m^6^A modified RNAs. (A)** IGV snapshots of positions predicted as m^6^A modified in 100% m^6^A curlcake. Individual reads are reported as “collapsed” and bases are colored by quality score, which contains information about m^6^A probability. The first two panels show reads from 2 independent m^6^A-modified curlcakes, and on bottom panels show individual reads from 2 independent unmodified curlcakes. The underlying reference sequence is shown in the bottom of each panel. The predicted m^6^A frequency by the *m^6^ABasecaller* for each site and replicate obtained with modification probability threshold of 0.1 is shown on the right of each panel. **(B)** Replicability between sites predicted in 2 replicates of sequencing of 100% m^6^A-modified curlcakes. **(C)** Replicability between predicted modification frequency in 2 replicates of sequencing of m^6^A-modified curlcakes (modification frequency predicted with modification probability threshold of 0.1, axes are log-scaled).


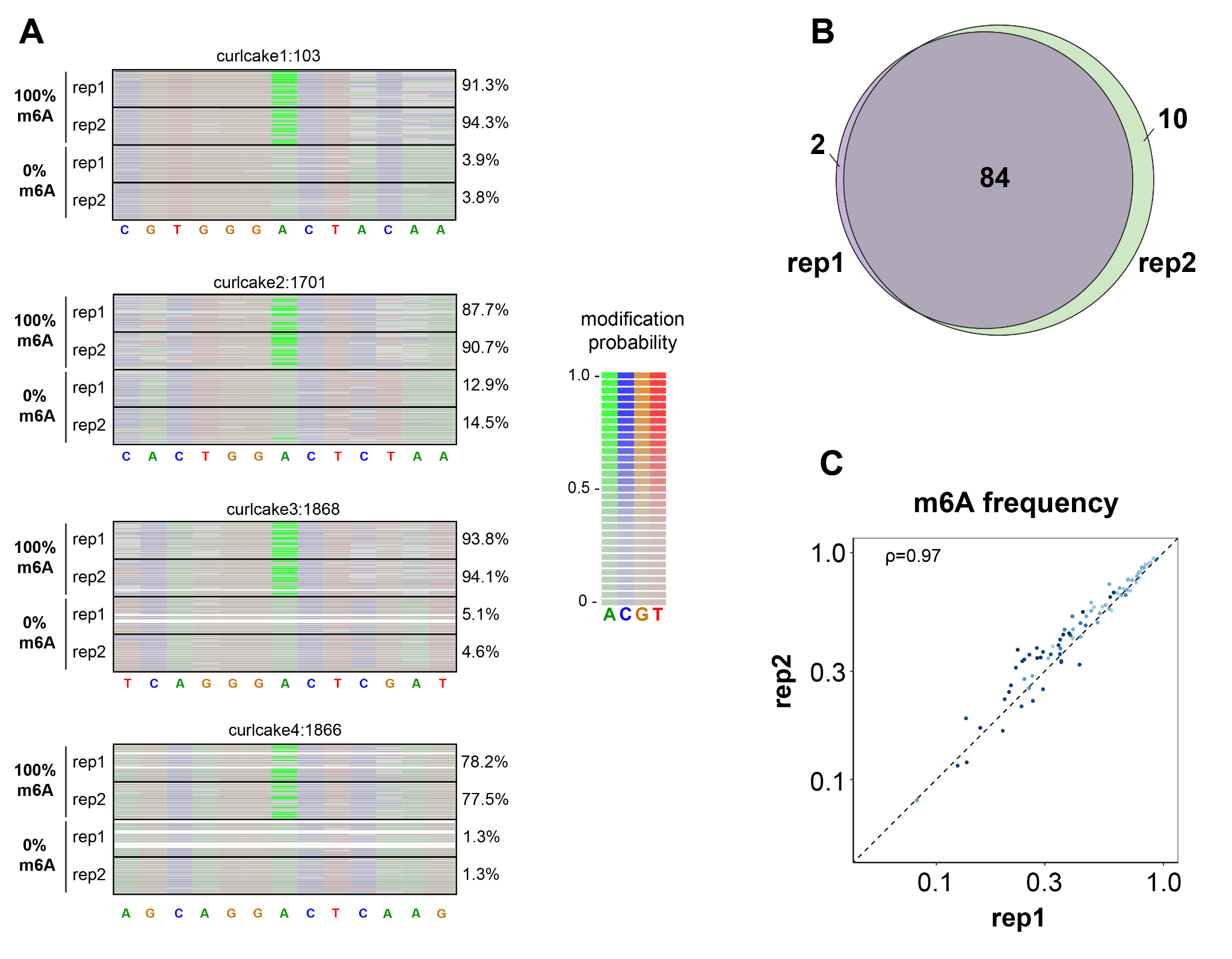


**Figure S6. Characterization of m^6^A sites predicted by *m^6^ABasecaller* in human (HEK293T), mouse (mESC) and zebrafish (4hpf embryos).**  **(A,B)** Metagene plot of the distribution of m^6^A sites along coding transcript features, in mESC (A) and zebrafish (B) publicly available DRS datasets. **(C,D)** Venn diagram depicting the intersection between predicted m^6^A sites in 2 independent biological replicates of HEK293T (C) and mESC (D) DRS datasets. A site was defined as “m^6^A-modified” if it had a minimum coverage of 25 reads and a modification stoichiometry greater or equal than 5%. **(E,F)** Scatterplots depicting the correlation of modification stoichiometry of m^6^A sites in HEK293T (E) and mESC (F) WT samples. Each dot represents an m^6^A site. Only m^6^A sites with more than 5% modification frequency and coverage greater or equal than 25 reads of coverage (in mESC, panel E) or 50 reads coverage (in HEK293T, panel F) in both replicates were included in the analysis. See also **Figure 3D** for scatterplot of modification frequencies across HEK293T WT replicates with 25 reads coverage threshold.


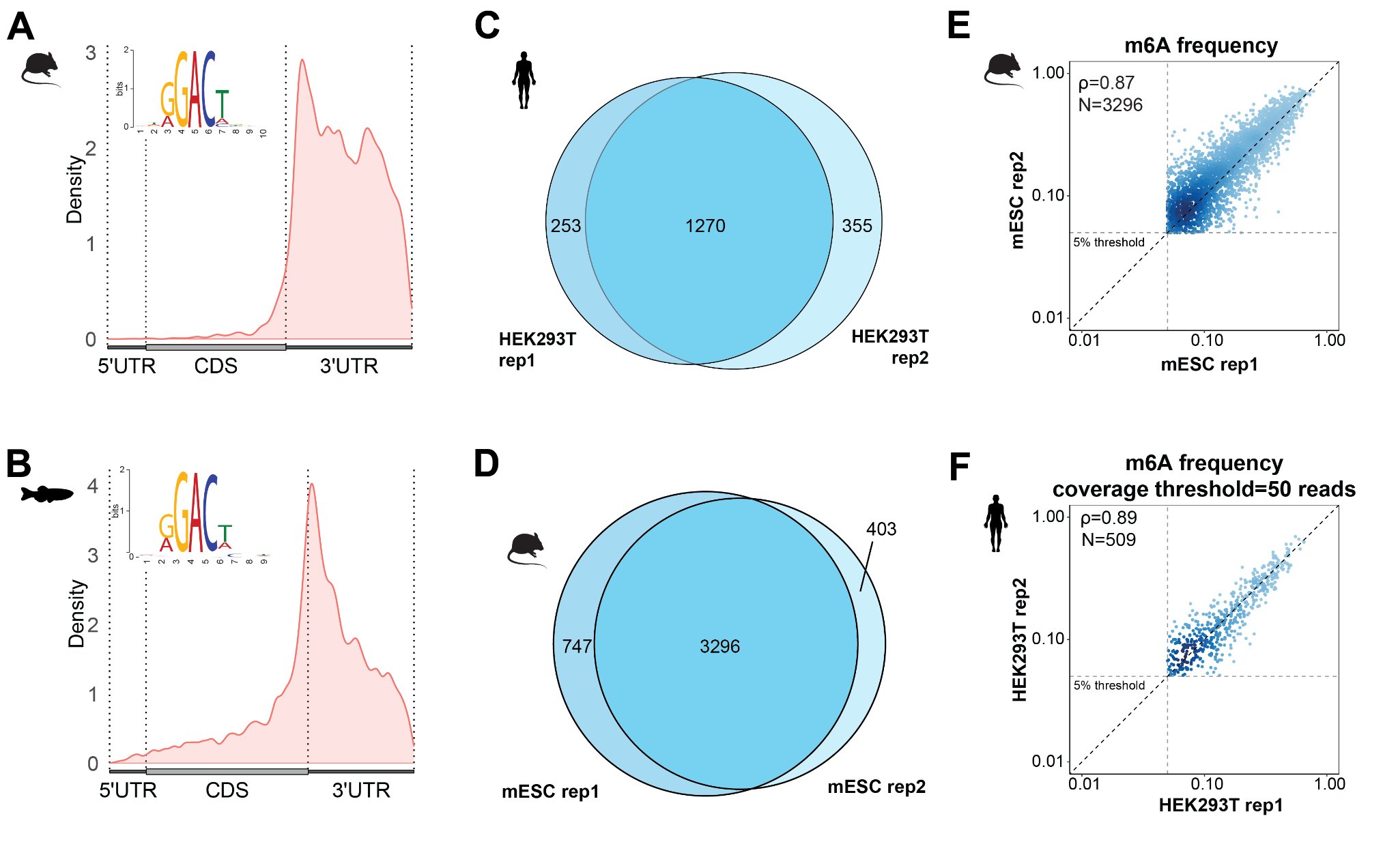


**Figure S7. *m^6^ABasecaller* captures quantitative changes in m^6^A stoichiometry in mES cells upon METTL3 or METTL14 KO. (A,B)** m^6^A modification frequencies in WT vs METTL3 KO (A) and in WT vs METTL14 KO (B) mESc DRS samples. Vertical and horizontal dashed lines denote the 5% threshold applied for a given site to be predicted as ‘m^6^A-modified’. Both axes are log_10_-scaled for enhanced visualization. **(C)** Density plot distribution of m^6^A modification frequencies in HEK293T WT, METTL3 KO and IVT samples, in two independent biological replicates**.** Dashed vertical lines represent the median m^6^A modification frequency observed in each sample (rep1 WT: 15.1%, rep2 WT: 15.6%, rep1 KO: 2.8%, rep2 KO: 2.9%, rep1 IVT: 0%, rep2 IVT: 0%). **(D)** Density plot distribution of m^6^A modification frequencies in mESC in WT, METTL3 KO and METTL14 KO samples**.** Dashed vertical lines represent the median m^6^A modification frequency observed in each sample (rep1 WT: 18.8%, rep2 WT: 16.4%, METTL3 KO: 4.7%, METTL14 KO: 7.8%). For C and D, a pseudocount of 0.001 was added to all values to allow logarithmic scaled axes.

***
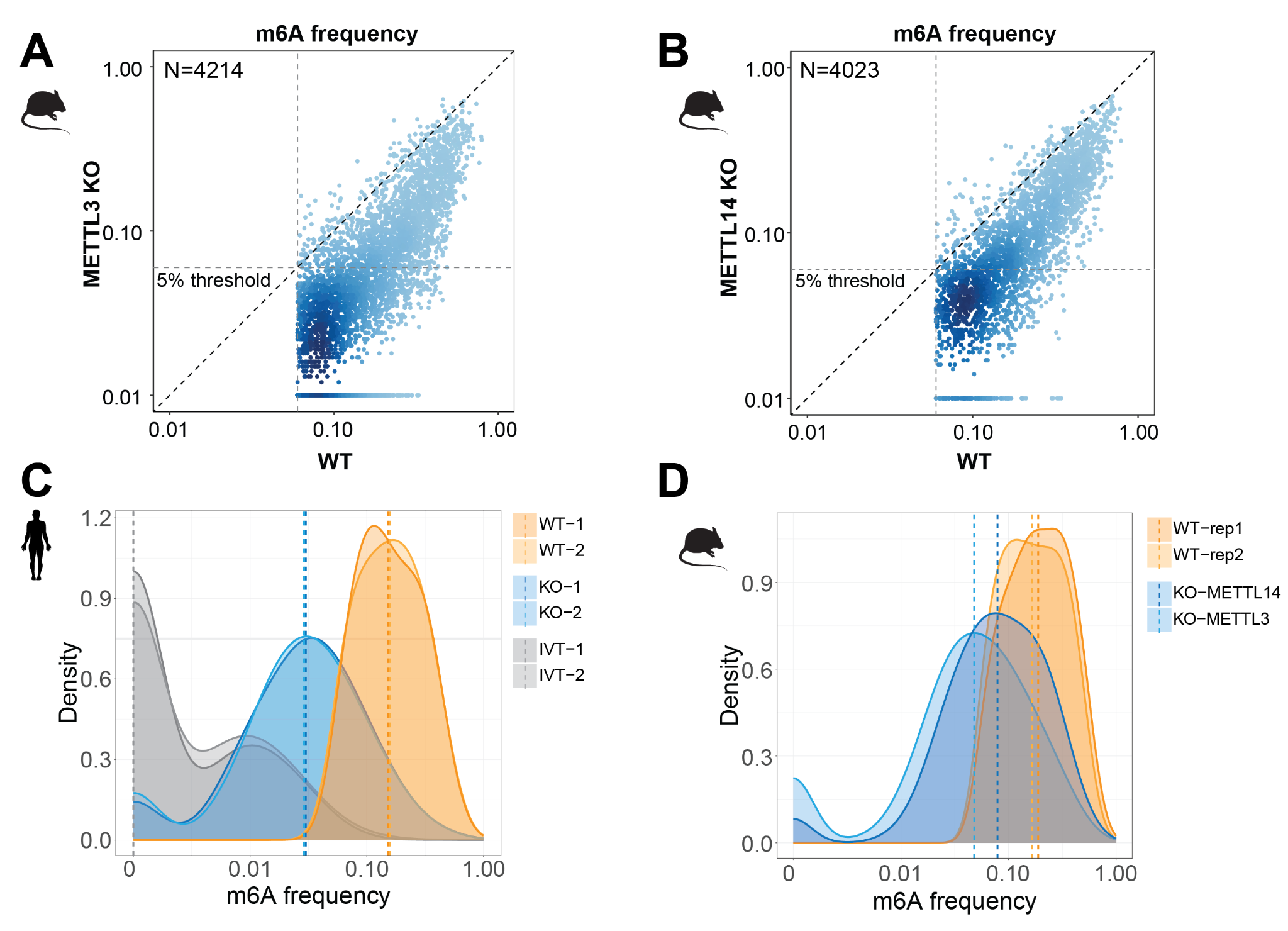
***

**Figure S8. Overlap between m^6^A sites predicted by *m^6^ABasecaller* and orthogonal methods in HEK293T cells. (A)** Overlap between m^6^A sites predicted by GLORIseq and *m^6^ABasecaller.* **(B)** Comparison of the predicted per-site stoichiometry using GLORI-seq and *m^6^ABasecaller*, in replicable sites that were predicted by both methods in HEK293T (n=2023 sites). A read was considered as m^6^A-modified if ModProb>0.1. Spearman’s ρ=0.73, p<2.2 e^-16^. **(C,D)** Overlap between m^6^A sites predicted by miCLIP and *m^6^ABasecaller* (C) and m6ACE-seq and *m^6^ABasecaller* (D) in HEK293T cells. **(E)** Overlap between predicted m^6^A sites using 3 different Illumina-based orthogonal methods (GLORI-seq, miCLIP and m6ACEseq) in HEK293T cells.

*
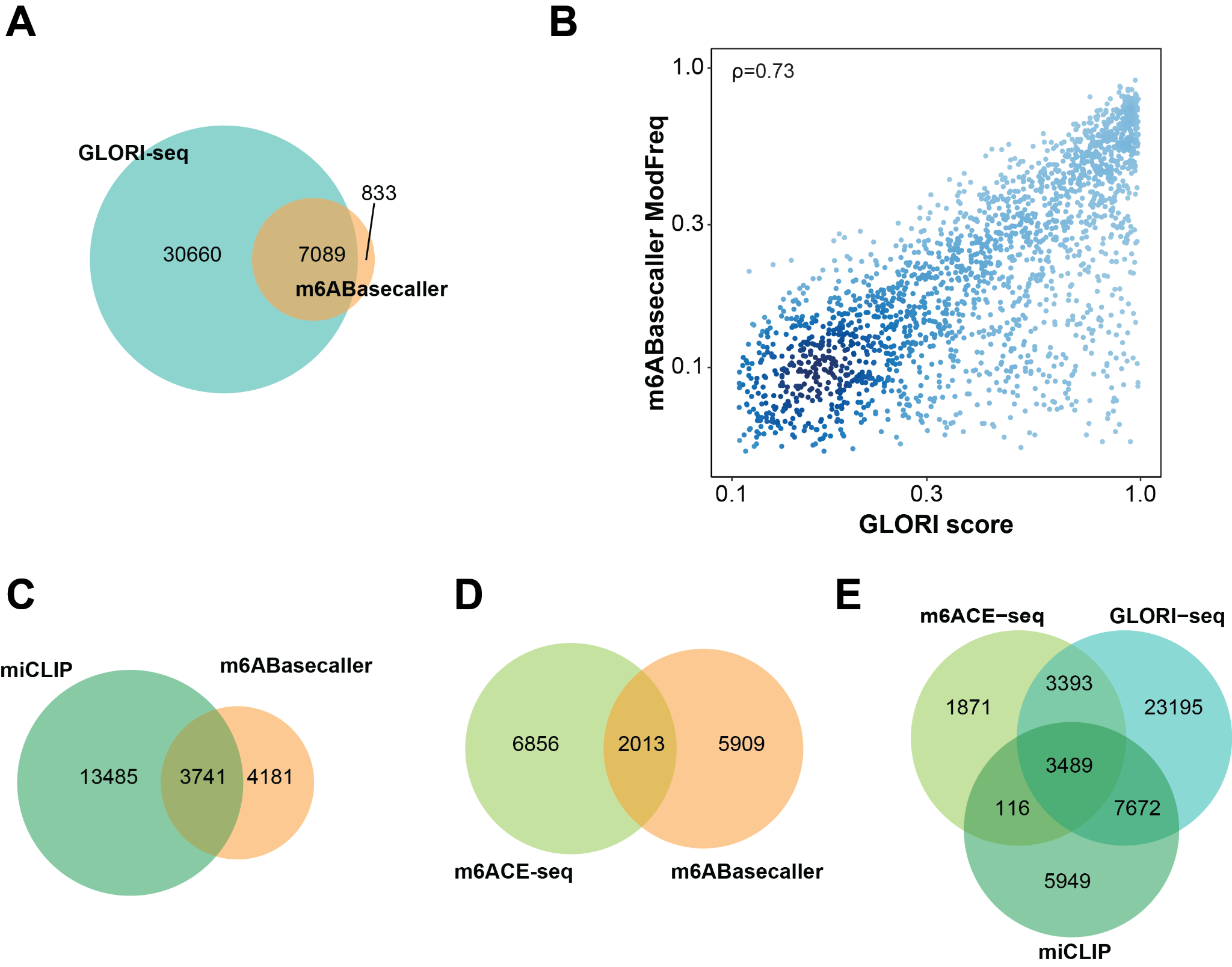
*

**Figure S9. Stoichiometry predictions in *in silico* mixtures with *m^6^ABasecaller* and m6Anet. (A,B)** Barplots showing the number of m^6^A sites predicted by *m^6^ABasecaller* (orange) and m6Anet (pink) in GGACT context (N=14 sites in curlcakes, marked by the horizontal dashed line, panel A) and in KGACY context (N=44 sites in curlcakes, marked by the dashed line, panel B) **(C,D)** Boxplot showing predicted m^6^A frequency for 0%, 6.2%, 12.5%, 25%, 50%, 100% m^6^A curlcakes in GGACT context (N=13 sites analyzed) (C) and in KGACY context (DRACH with motives with no more As in the fivemer, N=41 sites analyzed) (D) for *m^6^ABasecaller* (orange) and m6Anet (pink).

**
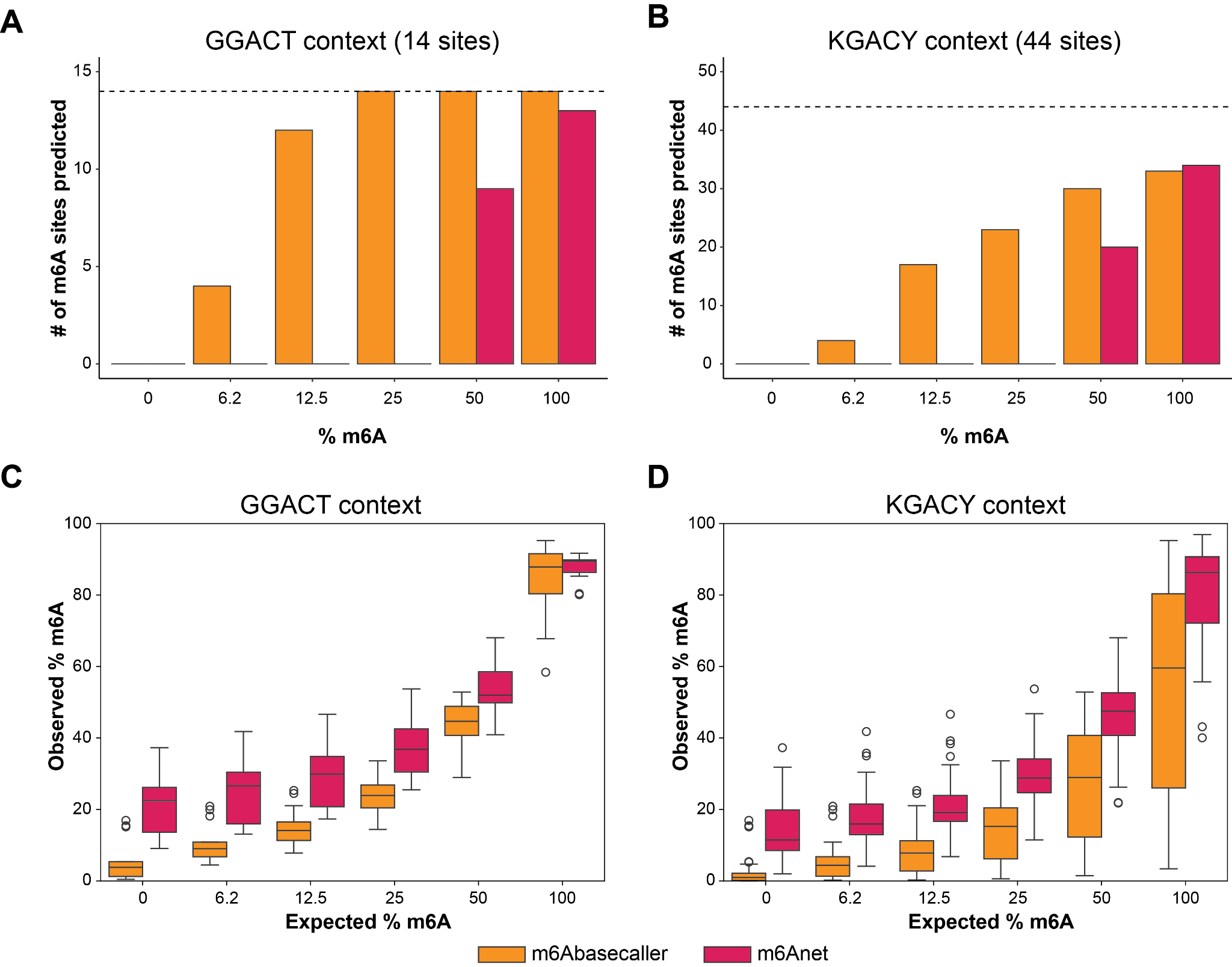
**

**Figure S10. Changes in m^6^A modification stoichiometry in mESC mRNAs upon STM2457 treatment. (A)** Euclidean distance matrix calculated on the modification frequencies measured when using different concentrations of STM2457 in 2 independent replicates. **(B)** Scatterplot depicting the replicability of m^6^A modification frequencies predicted, for untreated (CTR) and treated samples, with 3 different inhibitor concentrations: 2uM, 10uM and 20uM. Axes are log_10_-scaled. **(C)** MEME motif obtained using as input the sequence context of predicted m^6^A sites in mESC untreated (CTR) samples. **(D)** Metagene plot of the distribution along coding transcripts (N of genes=385) of the m^6^A sites found in CTR pooled samples (N of sites=584) with >=25 reads of coverage and >=5% frequency.

**
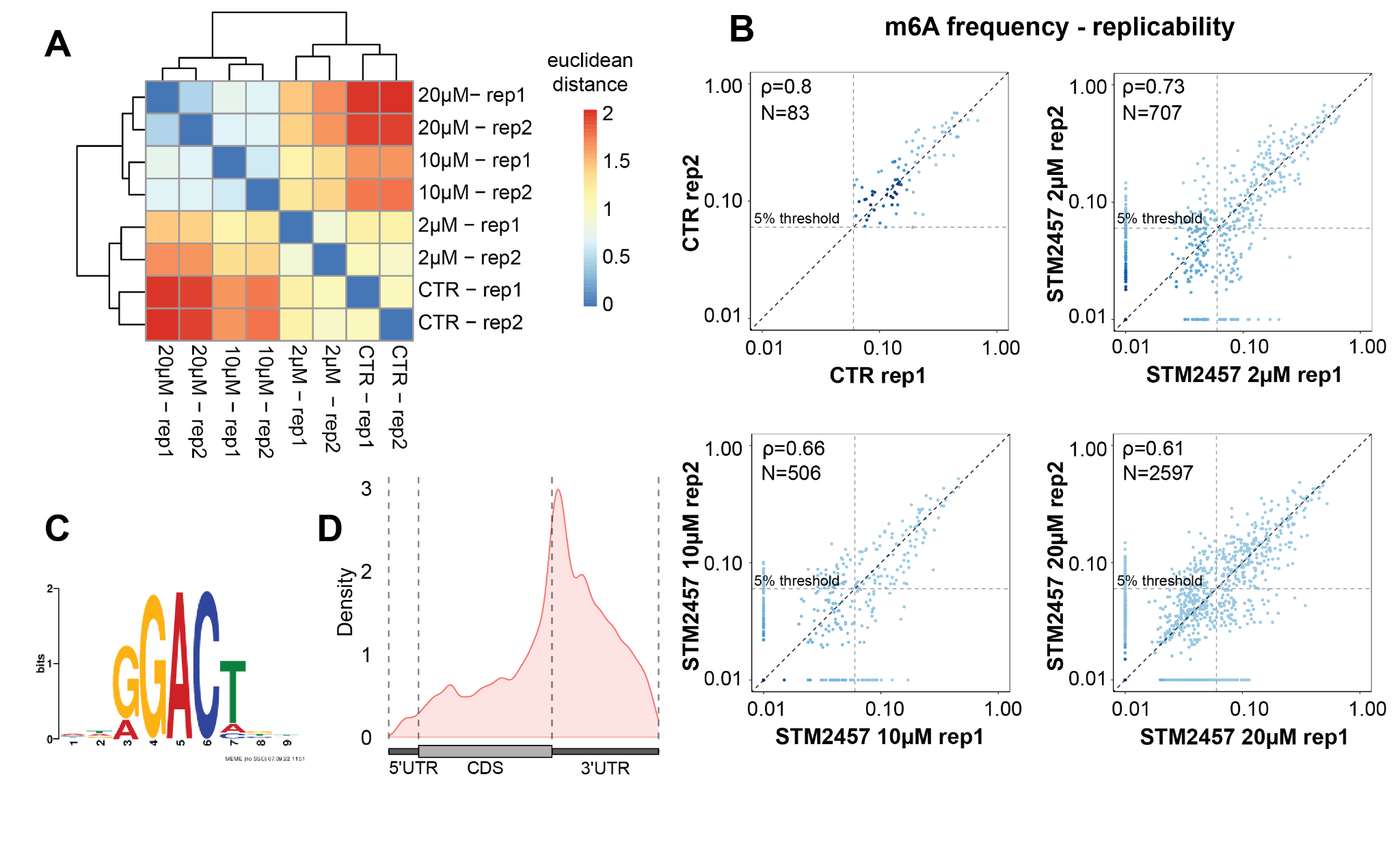
**

**Figure S11. Replicability of m^6^A frequencies in tamoxifen-treated and untreated mESC cells.** **(A)** Replicability of m^6^A frequencies in two replicates of mESC treated with tamoxifen (METTL3 KO) or vehicle MetOH (CTR) for 6 days. Both axes are log_10_-scaled. **(B)** Replicability of m^6^A frequencies in the two replicates of mESC cells treated with tamoxifen (KO) or vehicle MetOH (CTR) for 14 days. Both axes are log_10_-scaled.


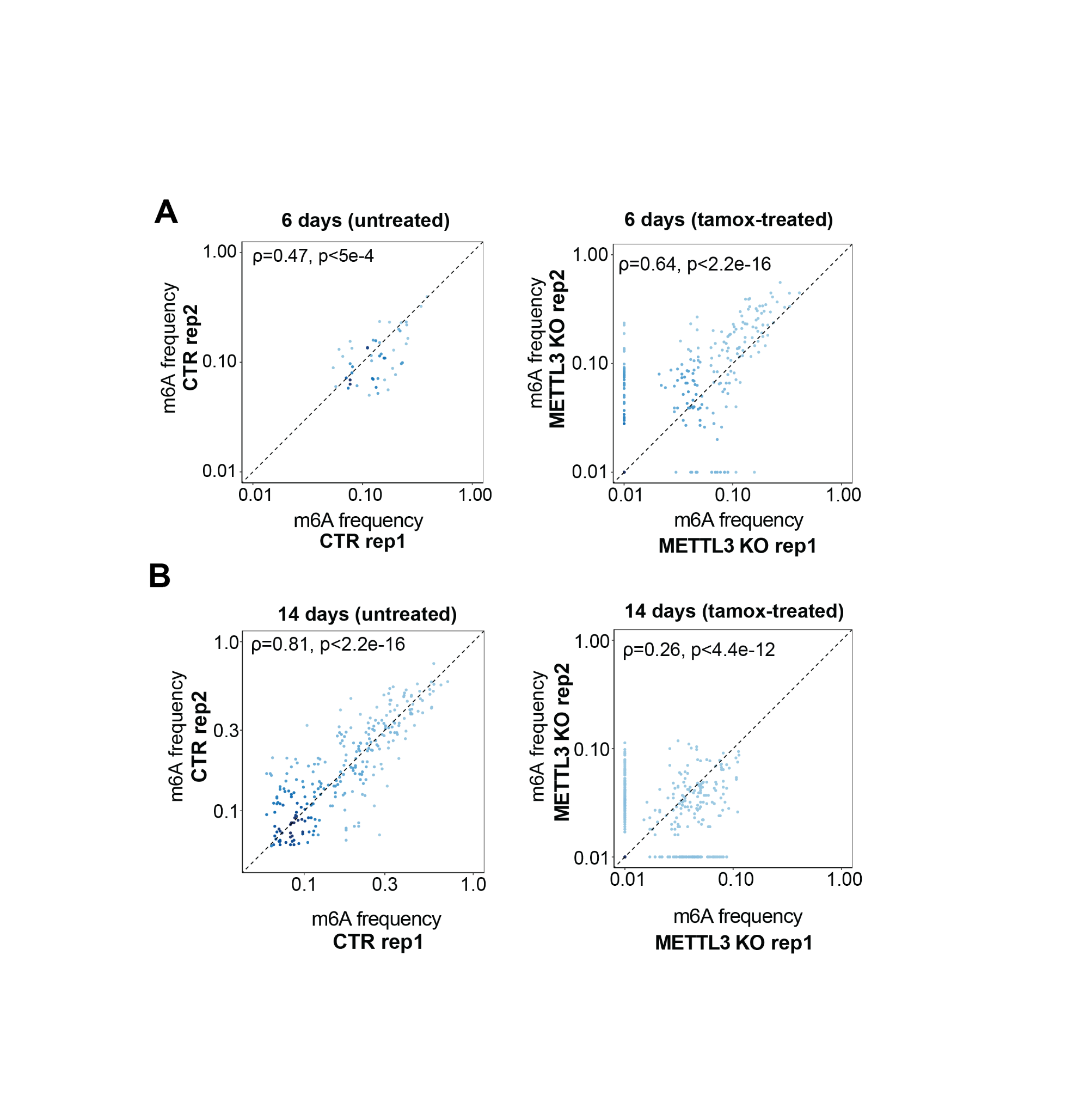


**Figure S12. Per-read analysis of m^6^A-modified sites in HepG2 cells shows dependency between m^6^A presence and polyA tail length. (A)** Density plot distribution of polyA tail length of reads that contain m^6^A (red) versus the one of reads that do not contain m^6^A (blue). Only reads mapping to genes that contain at least one m^6^A site have been considered in this analysis (n=442,126 reads, median polyA tail length: 84nt for no_m6A, 90nt for m6A, Mann-Whitney-Wilcoxon test p<2.2e^-16^). **(B)** Density plot distribution of median polyA tail length of reads that contain (red) or do not contain m^6^A (blue), considering only genes that had at least 10 or more m^6^A sites in their reads (n=1507 genes, median polyA tail length: 89nt for no_m6A, 92nt for m6A, Mann-Whitney-Wilcoxon test p<0.05).

**
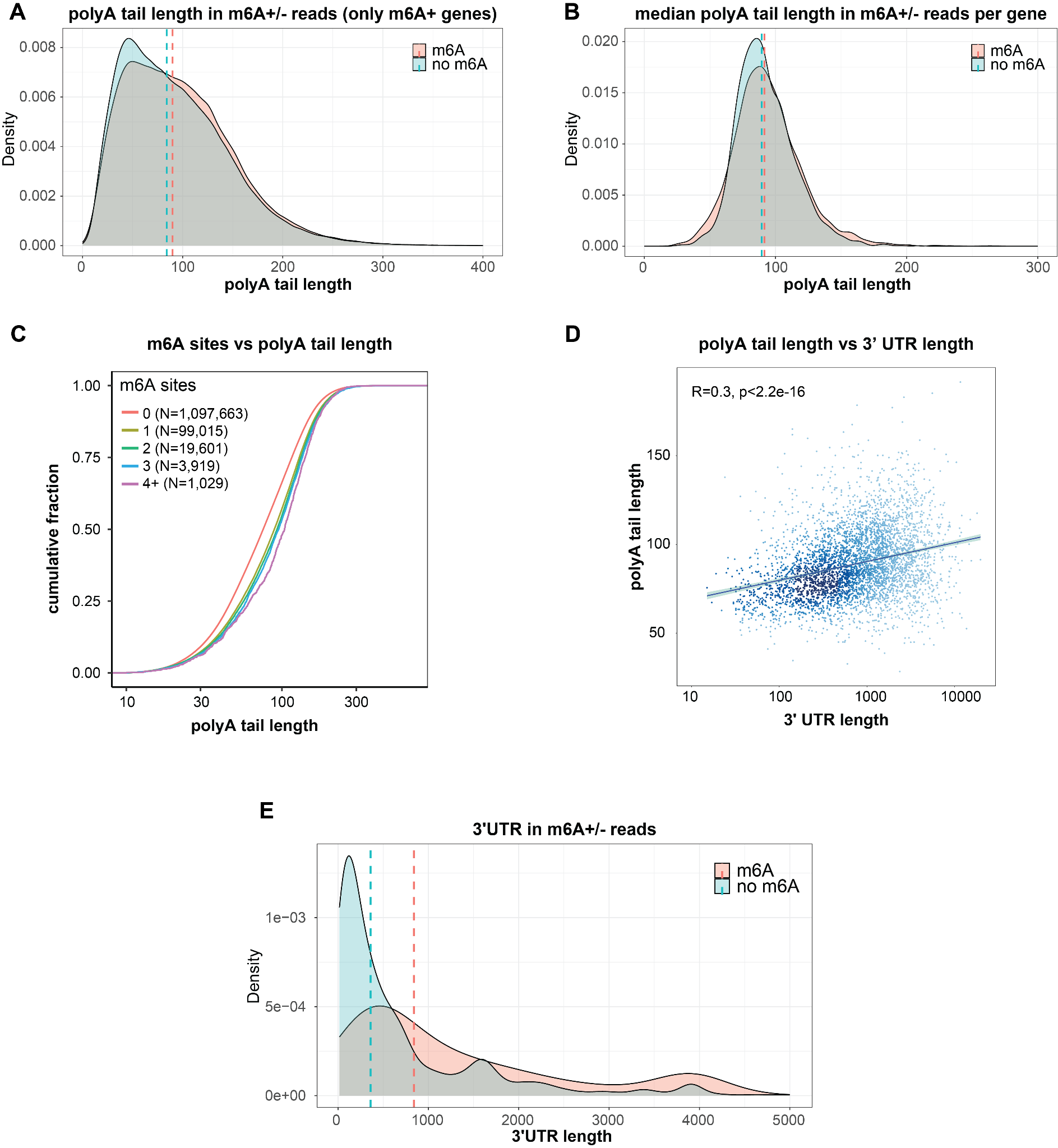
**

**Figure S13. Per-isoform analysis of m^6^A modifications. (A)** IGV snapshot of two isoforms belonging to gene UQCRFS1. In the zoomed section, the presence of m^6^A at per read level is shown with bright red color (as the reads map to the “-” strand). Clustering of reads belonging to reassigned isoforms ENST00000304863_0 (left) or ENST00000304863_1 (right) based on their modification pattern in positions chr19:29207544 and chr19:29207538. Next to both snapshots, results from the co-occurrence analysis are shown. **(B)** IGV snapshot depicting the modification frequency at position chr9:136,862,324 in two isoforms from gene EDF1. Modification frequency at per-isoform level is shown in two replicates.


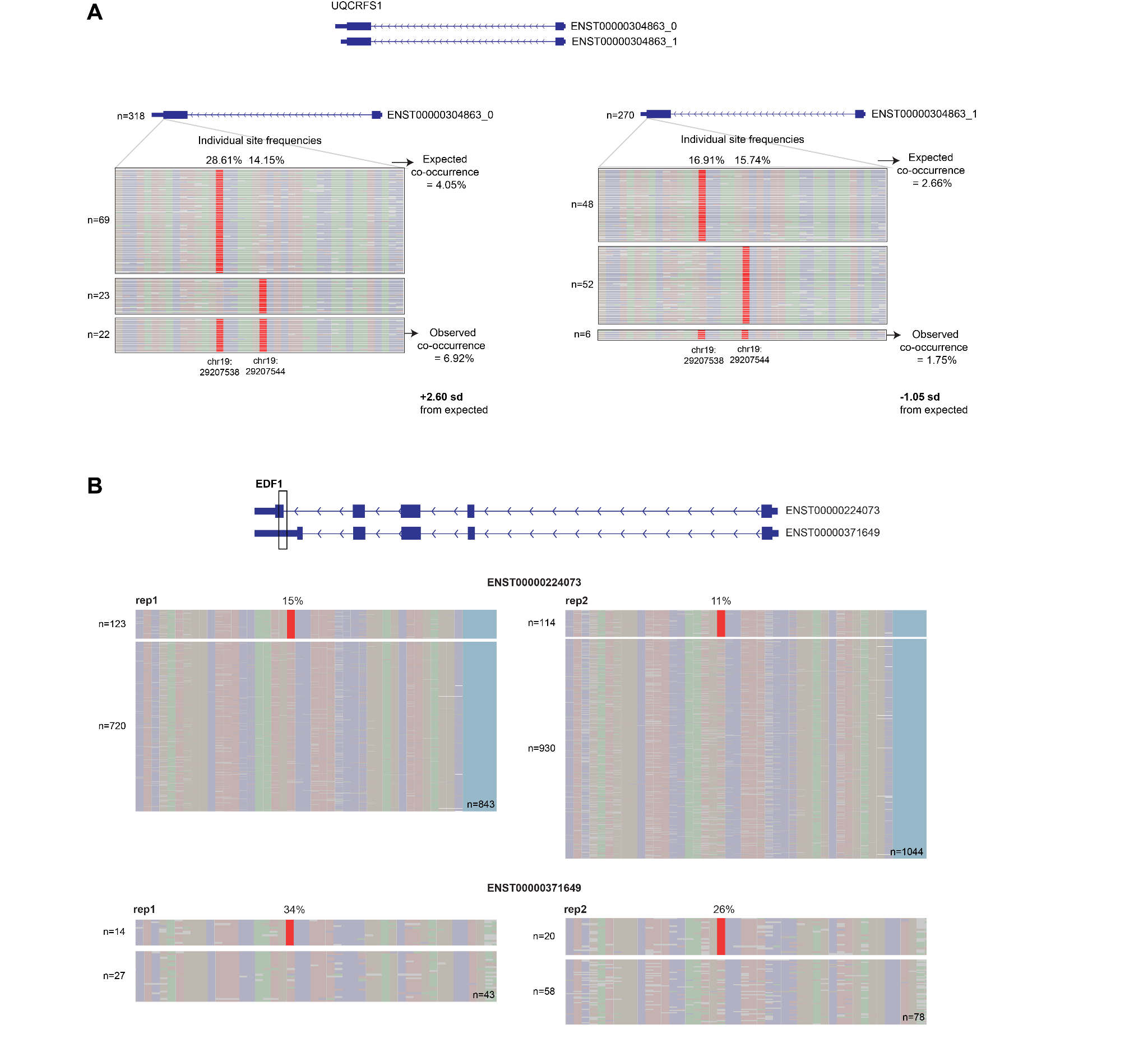


**Figure S14. m^6^A frequency variability between isoforms and distance from exon end. (A)** Scatterplot showing the difference in m^6^A frequency between two isoforms (y axis) and the absolute distance in nt of the m^6^A site from the closest exon end (x axis). A gradient from light to dark blue depicts the increase in density of data points in the plot. **(B)** Distribution of the distance of the m^6^A sites from the closest exon end, binned into “varying” and “non varying” based on whether the difference in m^6^A frequency between the two isoforms is >=5% or not, respectively.


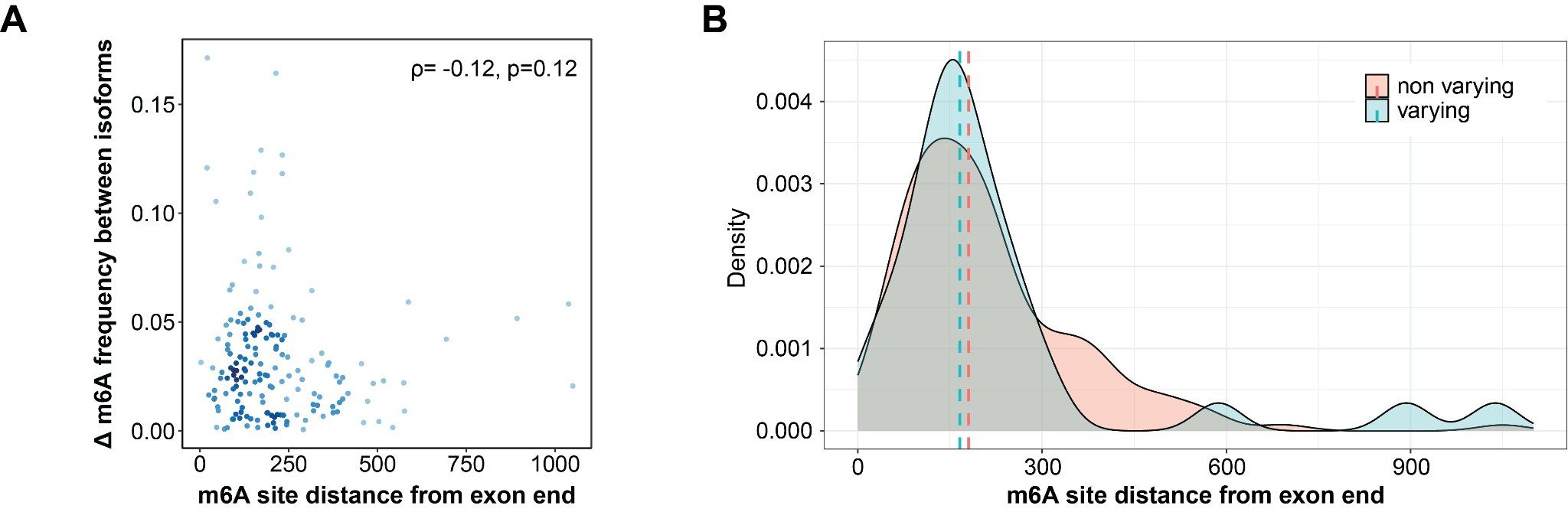


**Figure S15. Motifs encountered by NanoRMS2 across DNA and RNA datasets, when comparing WT with knockout/PCR/IVT datasets. (A)** Comparison of E.coli native DNA versus PCR-amplified DNA captures two motifs: GATC (which harbours m^6^A DNA modifications) and CCWGG (which is known to harbour m5C DNA modifications) **(B)** Comparison of Human native DNA versus PCR-amplified DNA captures CpG motif (which is known to harbour m^5^C DNA modifications). **(C)** Comparison of *S. cerevisiae* WT versus ime4 KO direct RNA sequencing data (data from [^1^](https://paperpile.com/c/jroQku/ee4U)) captures DRACH motif, with a slight preference towards GGACA. **(D)** Comparison of mESC wild type versus Mettl14 KO direct RNA sequencing data (data generated by [^2^](https://paperpile.com/c/jroQku/QEvh)) captures the DRACH motif (which is known to harbour m^6^A RNA modifications), with a slight preference towards GGACU, which is also reported when using other illumina-based methods.

*
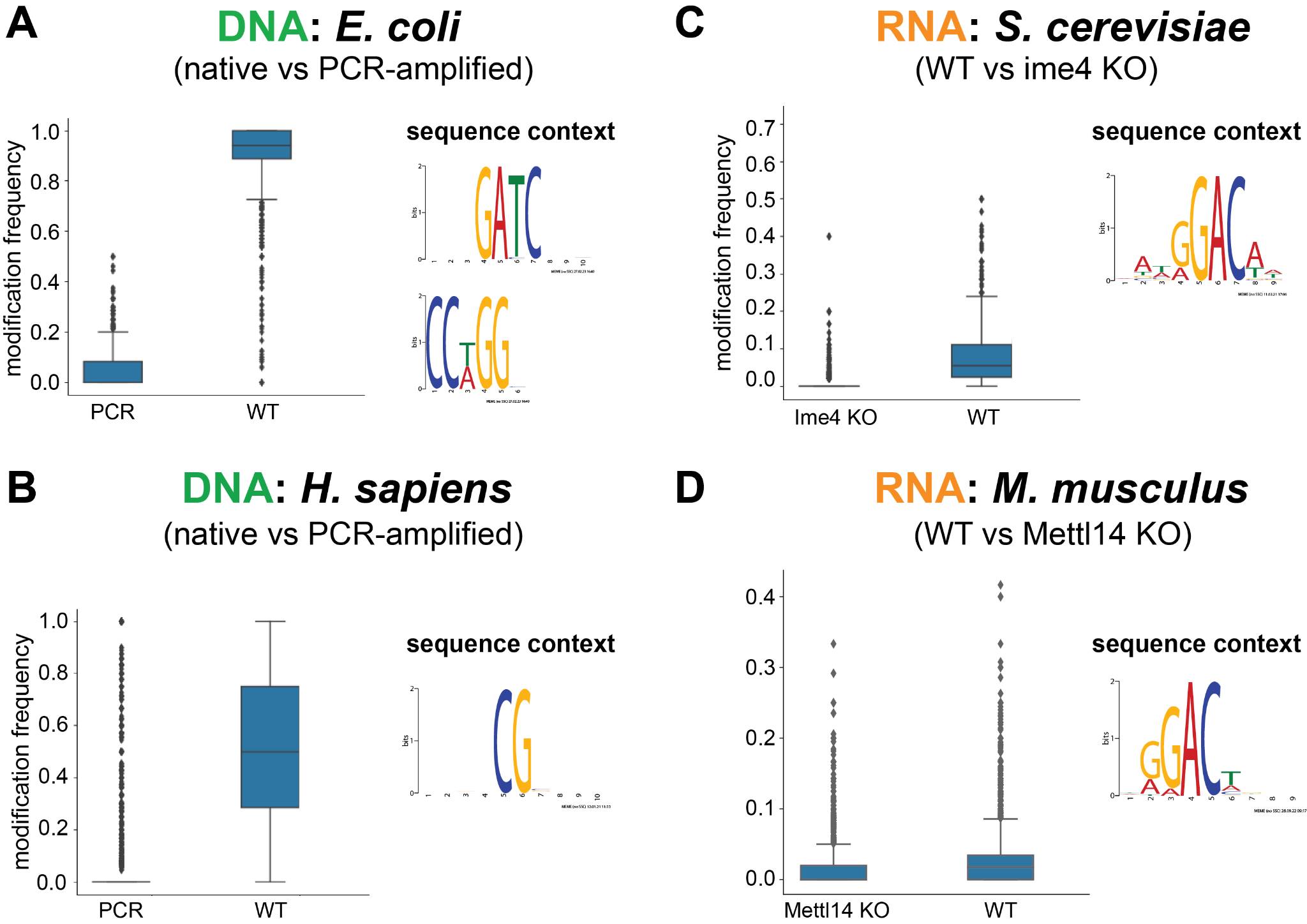
*

**Figure S16. Features extracted in modified and unmodified reads, sequenced with RNA004 chemistry.** Density plots of the Trace (TR) and mean Signal Intensity (SI) observed in a random subset of k-mers, both illustrating the values obtained for unmodified (blue) and modified (orange) reads, at positions -1, 0 and 1 (from left to right) of the k-mer. The results are shown for 3 distinct k-mers, containing either m^6^A at the middle position (**A**) or ac^4^C at the middle position (**B**). TR values were obtained using bonito v0.8.1 with rna004_130bps_hac@v5.1.0 (hac) base-calling model. SI values were retrieved using remora v2.1.3. The k-mer analyzed in each plot is shown on the Y-axis, and the distribution of the TR and SI features of modified and unmodified reads at positions -1, 0 and +1 is shown for each k-mer.


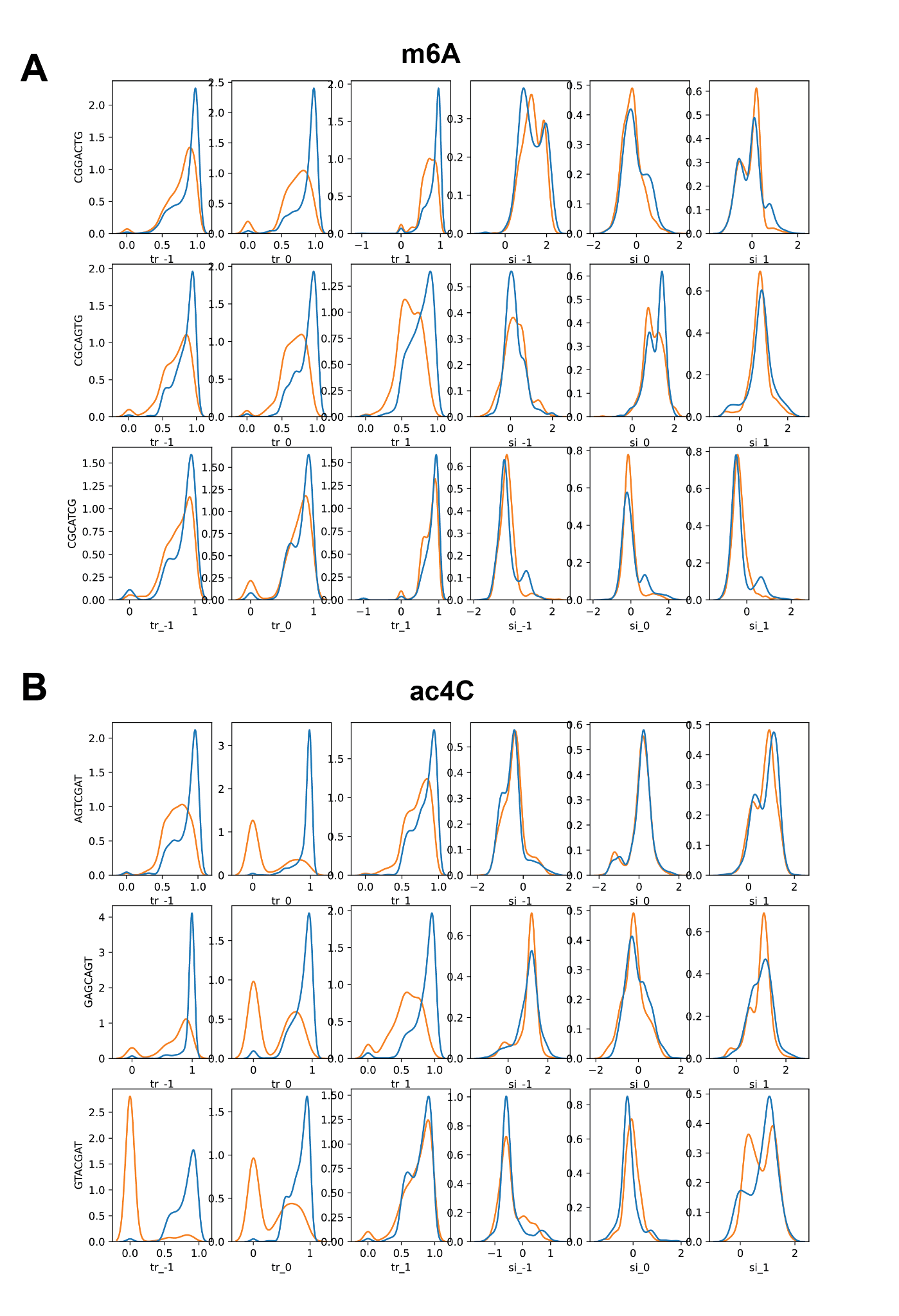


**Figure S17. IVT curlcakes produced with unmodified or 100% modified bases, sequenced with RNA004 chemistry.** IGV snapshot of curlcake IVT constructs generated with canonical or different modified nucleotides as input. (see **Table S2** for details). Reads were basecalled with dorado v0.8.0 using rna004_130bps_fast@v5.1.0 (fast) and aligned with minimap2 v2.28 using following parameters: -k13 -w4 -n1 -m15 -s30 -A1 -B1. Up to 1,000 reads with the longest alignment are shown for each sample. Allele frequency threshold is set to 0.2.


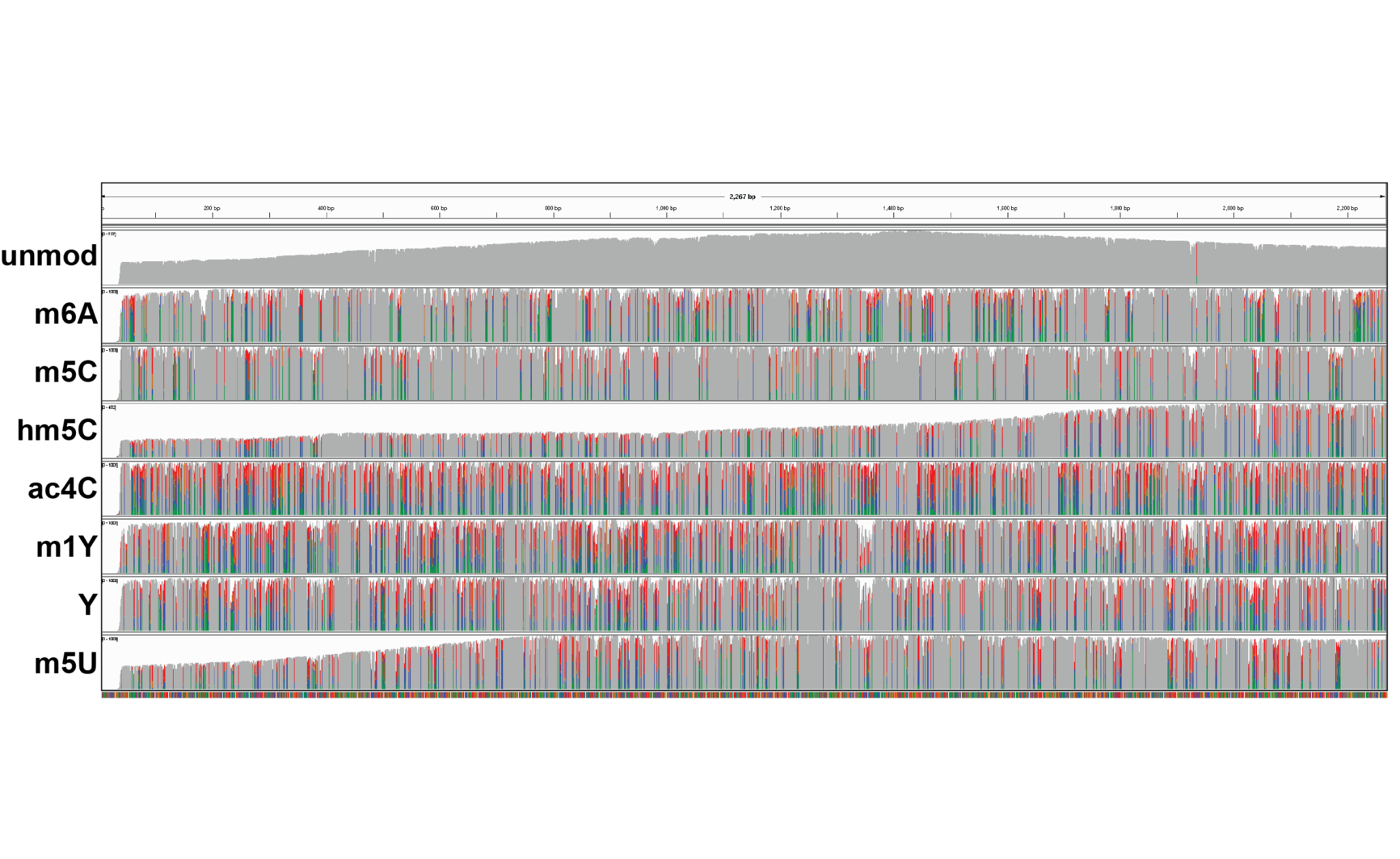


**Figure S18. Classification of RNA004 reads using the *hac* basecalling model. (A)** Receiver Operating Characteristic (ROC) curves, depicting the True Positive Rate vs False Positive Rate, using 39 features, and shown for 7 different RNA modifications (m6A, m5C, hm5C, ac4C, m1Ψ, Ψ and m^5^U). Gray lines represent individual k-mers, the blue line shows the mean between all k-mers. The number of positions included in the analysis and AUC is reported in the plot for each modification. Positions with fewer than 100 reads were discarded and not included in the plots below. **(B)** Comparison of ROC curves showing overall Random Forest Classifier performance per RNA modification type. AUC is reported for each modification.


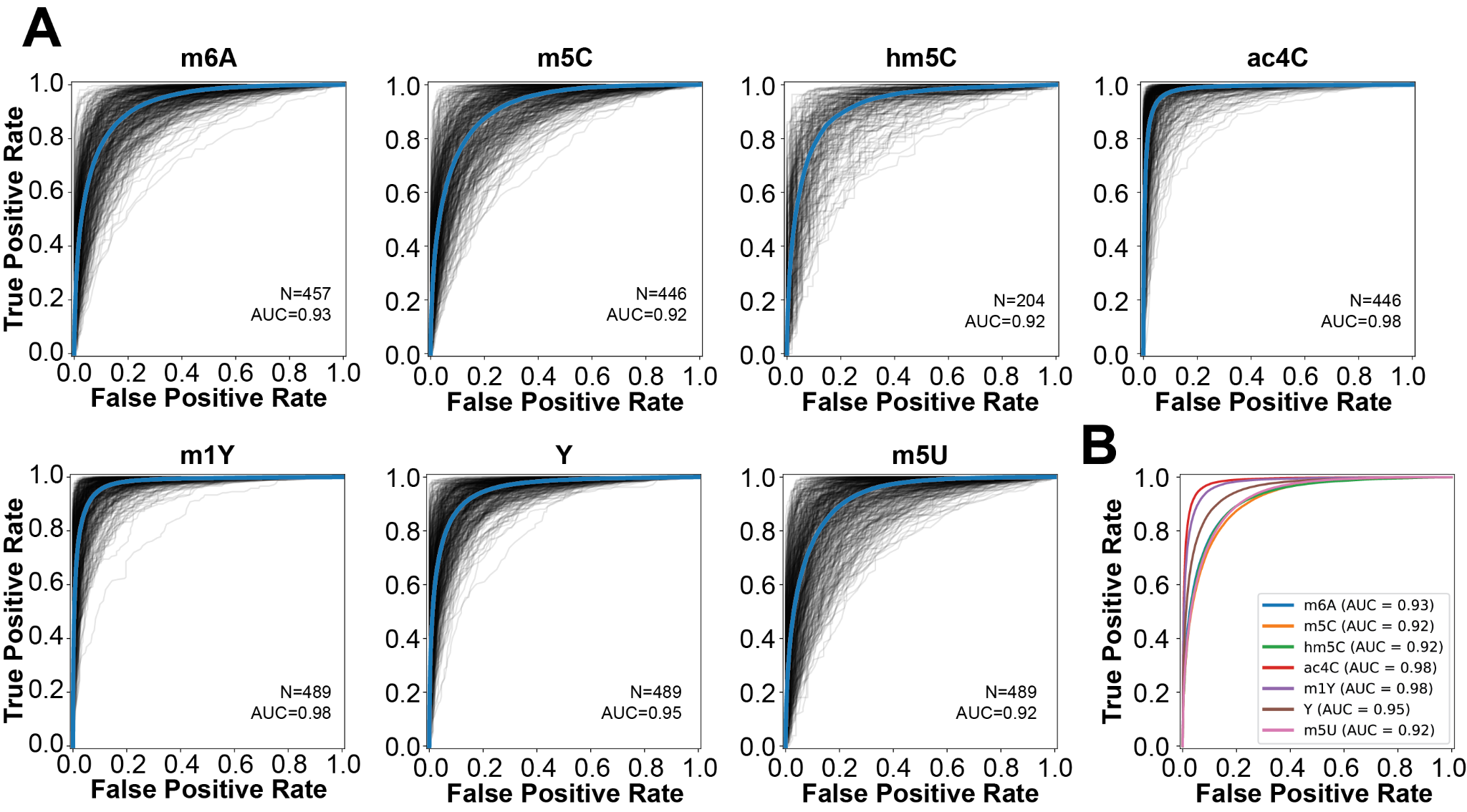


**Figure S19. Classification of RNA004 reads using the *fast* basecalling model. (A)** Receiver Operating Characteristic (ROC) curves, depicting the True Positive Rate vs False Positive Rate, using 39 features, shown for 7 different RNA modifications (m6A, m5C, hm5C, ac4C, m1Ψ, Ψ and m^5^U). Gray lines represent individual k-mers, the blue line shows the mean between all k-mers. The number of positions included in the analysis and AUC is reported in the plot for each modification. **(B)** ROC curves showing overall Random Forest Classifier performance per RNA modification. AUC is reported in the plot for each modification.

**
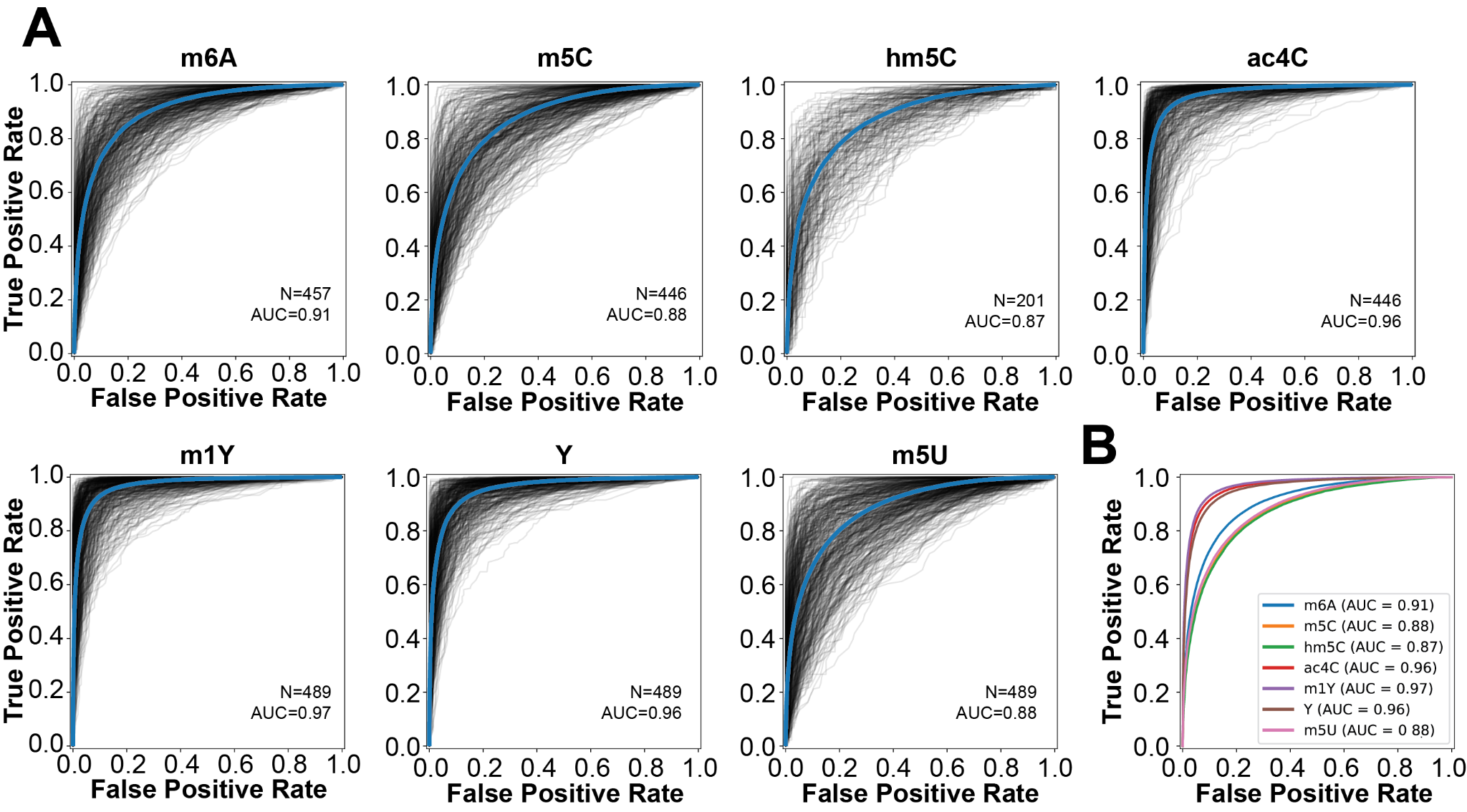
**

**Figure S20. Classification of RNA002 reads using the hac and fast basecalling model. (A,B)** ROC curves showing overall Random Forest Classifier performance per RNA modification, when reads were basecalled using *hac* (A) or fast (B) models. AUC is reported in the plot for each modification and basecalling model.


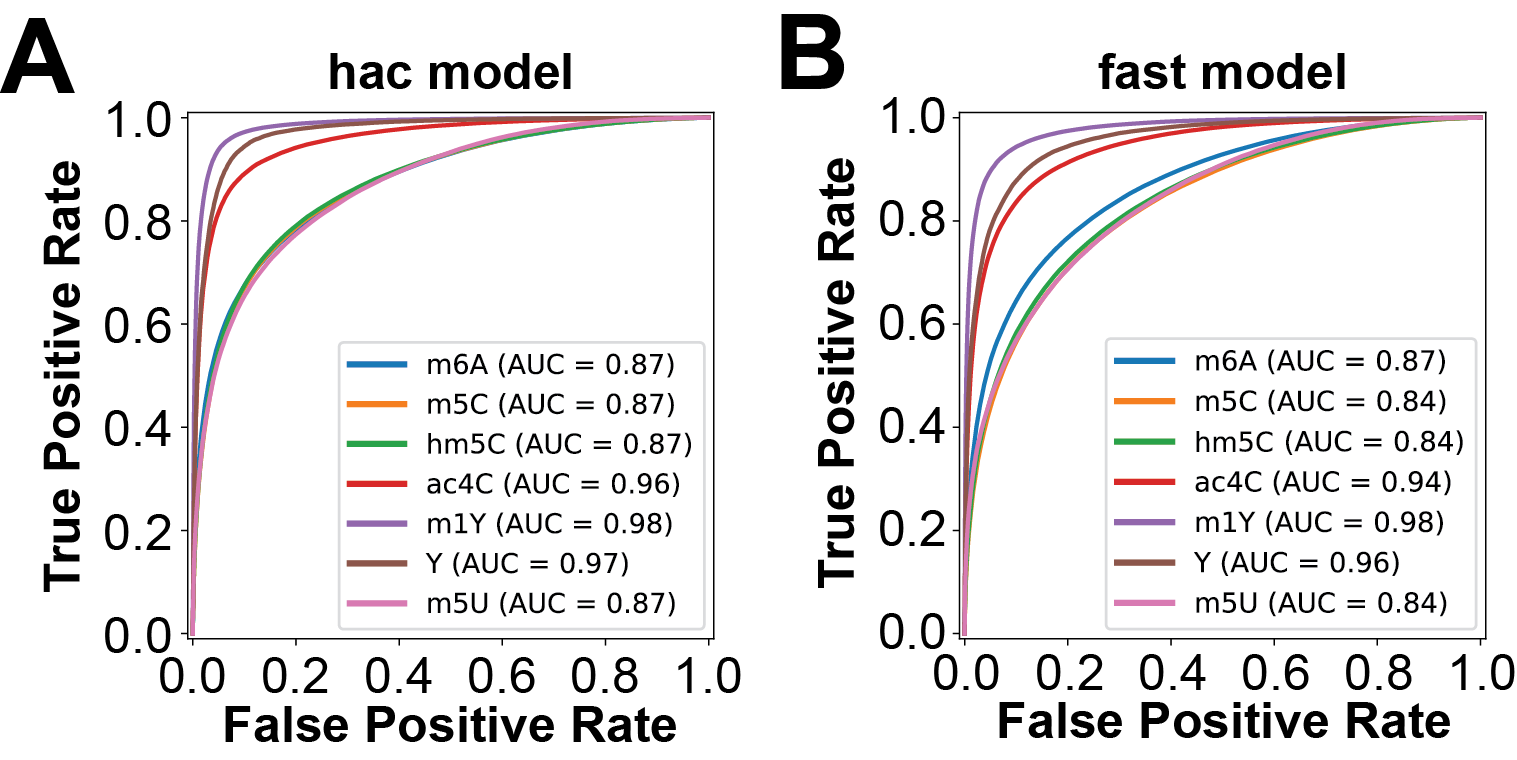

Supplement: Supplementary file 1 — Additional file 1. Contains all the supplementary Figures for this manuscript. [file 13059_2025_3498_MOESM1_ESM.docx]
